# Robust multimodal emotion recognition under incomplete modalities via transformer conditional variational autoencoders

**DOI:** 10.1016/j.isci.2026.117593

**Published:** 2026-09-23

**Authors:** Shuai Wang, Juan Li, Zhong Wu, Baoli Wang, Qi Wang

**Affiliations:** 1 School of Information Science and Engineering, Xinjiang College of Science & Technology, Korla 841000, China; 2 Shanxi Province Optoelectronic Information Science and Technology Laboratory, Yuncheng University, Yuncheng 044000, China; 3 School of Mathematics and Information Technology, Yuncheng University, Yuncheng 044000, China

**Keywords:** multimodal emotion recognition, incomplete multimodal learning, conditional variational autoencoder, transformer, robust representation learning, cross-modal fusion

## Abstract

Multimodal emotion recognition (MER) has gained increasing research interest owing to its ability to integrate complementary emotional cues from textual, acoustic, and visual modalities. However, real-world applications often suffer from incomplete multimodal inputs caused by sensor failures, communication disruptions, or noisy environments, resulting in missing modalities or corrupted features. To address these challenges, this study proposes RMM-TCVAE, a robust MER framework based on a Transformer-based Conditional Variational Autoencoder. The framework combines modality-specific encoders, a conditional Transformer Reconstruction Network for recovering missing modalities, and a Gated Multimodal Fusion Network for adaptive cross-modal integration. Experiments on CMU-MOSI, CMU-MOSEI, and IEMOCAP demonstrate that RMM-TCVAE consistently outperforms strong baselines under incomplete multimodal conditions, achieving 84.8% accuracy on CMU-MOSEI and yielding improvements of up to 2.9 percentage points in Acc and 3.1 percentage points in WAF across fixed missing-rate experiments. Ablation and visualization analyses support the effectiveness of RMM-TCVAE under incomplete multimodal conditions.

## Highlights

• A robust framework handles missing and corrupted multimodal inputs
• Transformer reconstruction recovers missing representations from available cues
• Conditional variational modeling captures uncertainty in missing modalities
• Reliability-aware fusion improves performance across three benchmark datasets

## Graphical abstract

**Figure:**
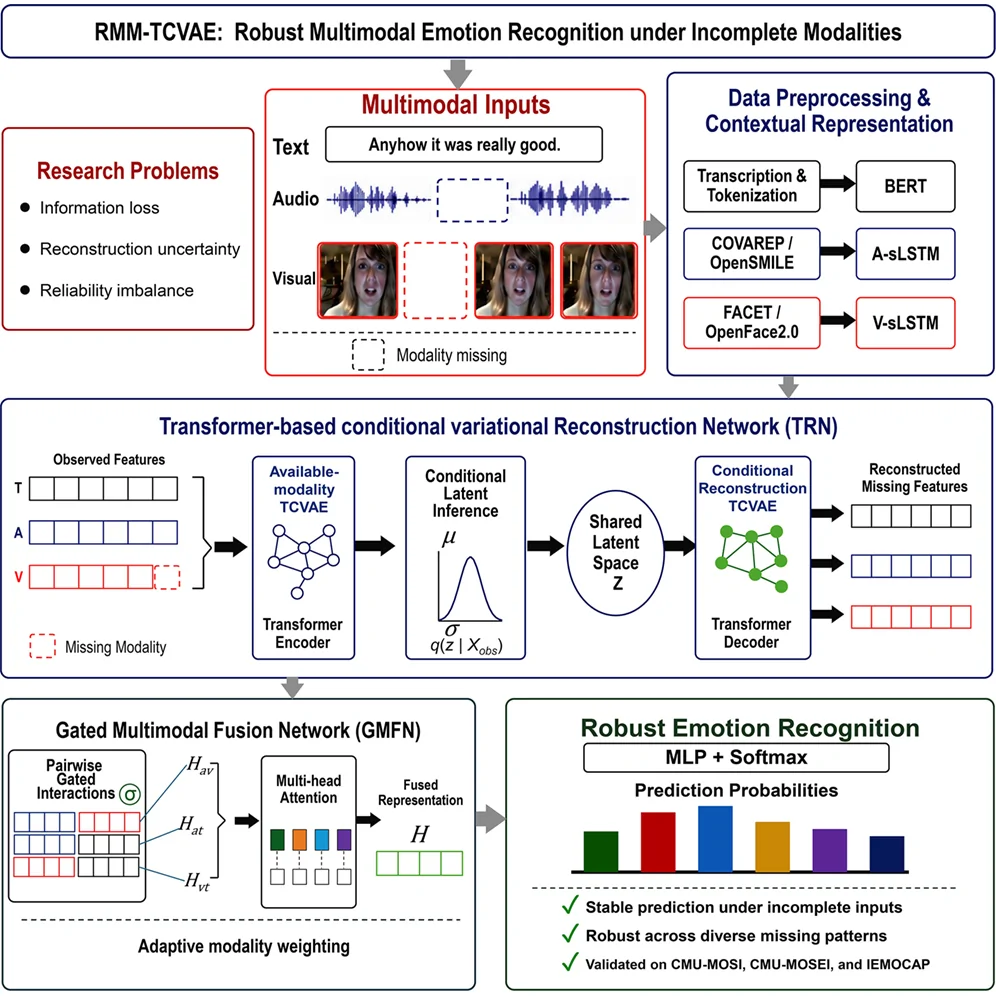

## Teaser

Applied sciences; Computer science; Computing methodology; Artificial intelligence; Emotion in artificial intelligence

## Introduction

Emotion recognition has long been a fundamental research topic in computer vision and artificial intelligence, aiming to understand human emotions from various data sources. In recent years, multimodal data have evolved into a primary channel for affective expression in both social media platforms (SMP) and human-computer interaction (HCI) systems. Such data typically encompass heterogeneous modalities, including textual content, acoustic signals, and visual cues, which exhibit rich semantic and emotional correlations. Accurate and robust emotion recognition has proven to play a key role in HCI, customer opinion mining, SMP, and decision support.^1^^,^^2^^,^^3^

By jointly modeling complementary signals from text, audio, and video, multimodal emotion recognition (MER) seeks to accurately identify human emotional states and has achieved substantial improvements over unimodal approaches. The integration of cross-modal information enables models to capture nuanced emotional cues that cannot be reliably inferred from a single modality alone.^4^^,^^5^^,^^6^ In the past few years, effective MER models have been proposed based on recurrent neural networks (RNNs), transformers, and graph neural networks (GNNs), achieving promising results under laboratory conditions. Existing approaches attempt to address multimodal learning through various fusion strategies.^7^ Early methods rely on feature concatenation or shallow fusion techniques, combining modality-specific representations at input or decision levels. More advanced architectures, including tensor fusion networks^8^ and attention-based fusion mechanisms,^9^ aim to model higher-order cross-modal interactions.

However, most existing MER models implicitly assume that all modalities (text, audio, video) are always available during both training and inference. In practical scenarios, missing modalities may arise due to sensor malfunction, data transmission errors, environmental noise, or user-selective input behavior. Such incomplete observations introduce cross-modal feature inconsistency and distribution shift, leading to severe performance degradation.^10^^,^^11^^,^^12^ This incompleteness manifests in two forms: (1) modality-level missing, where an entire modality is absent; and (2) feature-level corruption, where partial information within a modality is masked or noisy. These two types are rarely addressed together in existing literature. Unlike controlled laboratory settings, real-world multimodal systems must operate under uncertain and dynamically changing missing conditions. Therefore, developing robust MER models capable of handling random modality absence and feature-level corruption is both practically important and theoretically non-trivial. Figure 1 illustrates typical missing-modality scenarios encountered in real-world scenes. Missing modalities may result in severe information loss, reconstruction uncertainty, and reliability imbalance among modalities, posing significant issues to robust emotion recognition.

**Figure 1:**
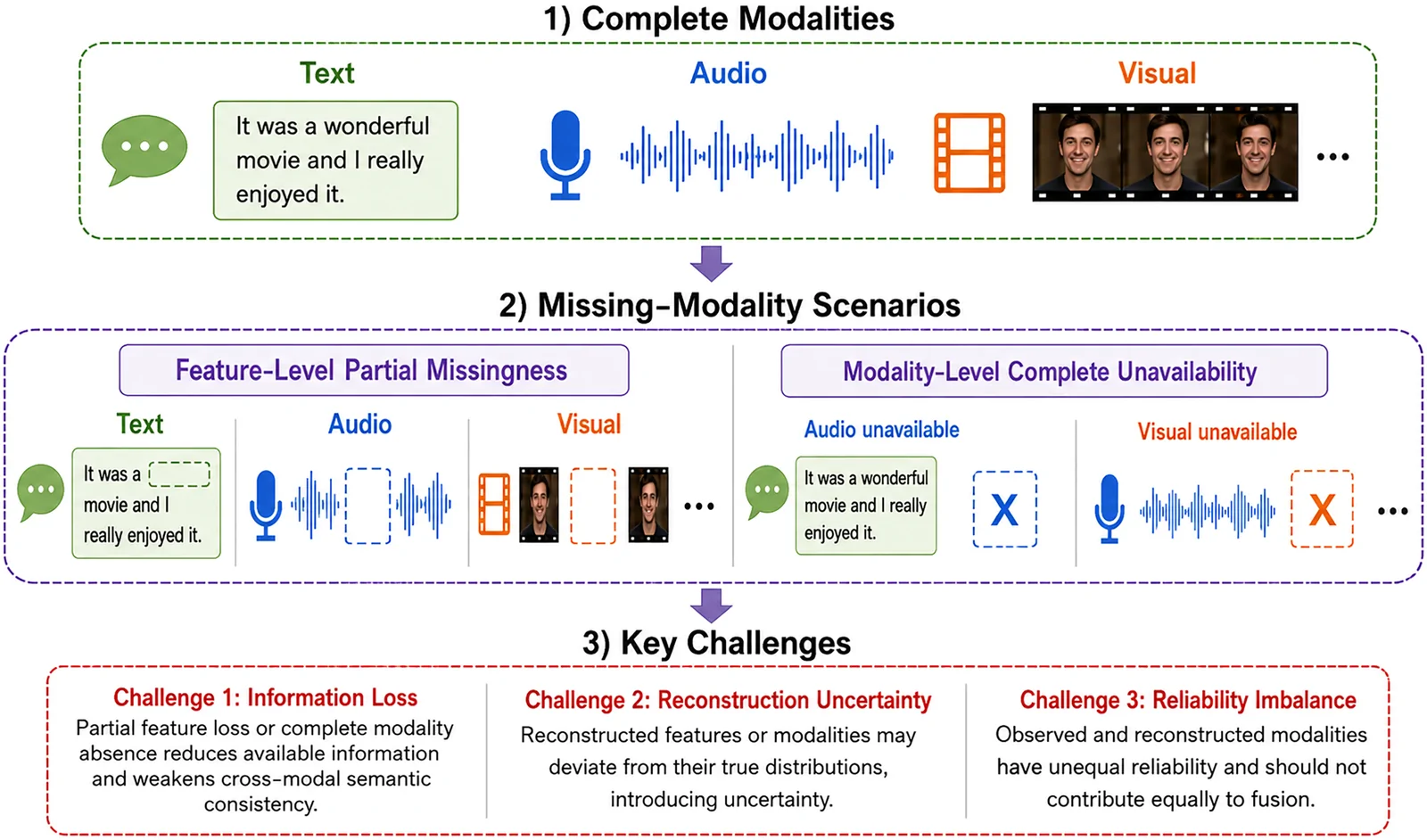
Missing-modality scenarios and key challenges in multimodal emotion recognition Complete multimodal inputs comprising text, audio, and visual information are contrasted with two representative incomplete-data scenarios: feature-level partial missingness and modality-level complete unavailability. Dashed boxes indicate missing features, whereas crosses indicate unavailable modalities. These conditions can lead to information loss, reconstruction uncertainty, and reliability imbalance between observed and reconstructed modalities.

Prior studies typically adopt either direct prediction based on available modalities or data imputation strategies.^13^^,^^14^ Early approaches often apply simple filling techniques, such as zero-padding or mean substitution, which ignore the underlying data distribution and semantic coherence, leading to feature distortion and bias. More recent methods attempt cross-modal translation to generate missing modalities from observed ones.^15^ However, cross-modal translation is inherently asymmetric and modality-dependent.^16^ While textual reconstruction may benefit from strong semantic priors and pretrained language models, generating reliable acoustic or visual representations from heterogeneous modalities remains significantly more challenging and is prone to noise accumulation. Furthermore, many existing approaches rely on fixed reconstruction patterns and assume predefined missing configurations.^17^^,^^18^^,^^19^ Under random multi-modality missing scenarios, these methods often introduce semantic ambiguity and fail to maintain cross-modal consistency, resulting in degraded robustness and generalization performance. Variational Autoencoders (VAEs) enable structured latent variable learning and uncertainty modeling, while conditional VAE extensions allow available modalities to guide reconstruction of missing components.^20^^,^^21^ In multimodal settings, joint generative models can align heterogeneous modalities within a shared latent space, facilitating cross-modal inference under incomplete conditions.

To address the missing-modality problems, a growing body of research has explored robust multimodal emotion recognition under incomplete observations. Existing methods can generally be categorized into four groups. First, cross-modal translation approaches, such as MCTN,^15^ reconstruct missing modalities through cyclic modality translation and shared latent representation learning. Second, reconstruction-based methods, including MMIN^13^ and IF-MMIN,^14^ employ modality imagination mechanisms to recover missing information from observed modalities. Third, graph-based and structure-aware methods, such as GCNet,^17^ model inter-modal relationships through graph completion to enhance representation robustness. More recently, noise-aware and uncertainty-aware frameworks, including NIAT,^19^ GMD,^22^ LNLN,^18^ and ROSA,^23^ have been proposed to improve robustness under random missing patterns and corrupted multimodal inputs. Liu et al.^24^ and Sun et al.^25^ developed modality translation and similarity completion strategies specifically for multimodal sentiment analysis under uncertain missing modalities. Figure 1 illustrates typical missing-modality scenarios in MER and highlights three key challenges, namely information loss, uncertainty in reconstructed representations, and reliability imbalance among modalities. Despite considerable progress in MER under incomplete data, the following challenges remain prevalent and can be summarized as follows:

• Accurately reconstructing missing modalities under high missing rates remains difficult because severe information loss weakens cross-modal semantic consistency.
• Existing works perform deterministic reconstruction and fail to explicitly model the uncertainty associated with missing-modality conditions. Consequently, reconstructed representations may not adequately capture the diverse latent distributions of missing information.
• Existing fusion strategies usually treat all modalities equally, which may introduce noisy or unreliable information into the final emotion prediction process.

These limitations significantly restrict the robustness and generalization capability of current MER systems. Therefore, the central research problem addressed in this work is how to simultaneously reconstruct missing modalities, model uncertainty in reconstructed representations, and perform reliability-aware multimodal fusion under missing modality conditions. Solving this problem is essential for achieving robust MER in real-world environments where modality availability cannot be guaranteed.

To tackle these challenges, we propose RMM-TCVAE, a Robust Multimodal Emotion Recognition framework under Missing Modalities via Transformer-Based Conditional Variational Autoencoders. The proposed architecture integrates modality-specific encoders, a cross-modal Transformer fusion module, and a conditional variational decoder. By leveraging available modalities as conditional guidance, RMM-TCVAE reconstructs representations of missing modalities while preserving cross-modal semantic consistency in the latent space. The incorporation of Transformer-based contextual modeling further enhances cross-modal dependency learning, enabling effective mitigation of feature inconsistency and performance degradation under diverse missing configurations.

The main contributions of this work are summarized as follows.

(1) To alleviate information loss and cross-modal inconsistency caused by random modality absence, we propose a Transformer Reconstruction Network (TRN) that exploits long-range dependencies among available modalities to reconstruct missing modality representations. By leveraging Transformer-based cross-modal interactions, the proposed TRN effectively enhances reconstruction quality and preserves semantic consistency across modalities.
(2) To address the uncertainty inherent in missing-modality reconstruction, we introduce a Transformer Conditional Variational Autoencoder (TCVAE) that performs conditional latent inference guided by available modalities. Unlike deterministic reconstruction approaches, TCVAE explicitly models latent uncertainty and learns modality-consistent latent representations, thereby improving the robustness and reliability of reconstructed features under incomplete observations.
(3) To mitigate the reliability imbalance between observed and reconstructed modalities, we develop a Gated Multimodal Fusion Network (GMFN) that dynamically adjusts modality contributions according to their inferred informativeness. This reliability-aware fusion strategy enables more effective integration of heterogeneous modalities and improves emotion recognition performance under diverse missing-modality scenarios.
(4) To validate the effectiveness and generalization capability of the proposed framework, we conduct extensive experiments on three benchmark datasets, namely CMU-MOSI, CMU-MOSEI, and IEMOCAP, under multiple missing rates and missing patterns. Experimental results demonstrate that RMM-TCVAE consistently outperforms several state-of-the-art missing-modality MER methods, while ablation studies further verify the contribution of each proposed component.

### Variational autoencoders

Variational autoencoders (VAEs) have emerged as a powerful class of generative models for learning structured latent representations and modeling complex data distributions.^26^^,^^27^ Since their introduction, VAEs have been widely adopted in multimodal learning due to their ability to capture shared latent structures across heterogeneous modalities and to model uncertainty through probabilistic inference. In the context of MER, VAEs provide a principled framework for representing correlated signals from text, audio, and visual inputs within a unified latent space.

The fundamental idea of VAE is to introduce latent variables to model the underlying generative process of observed data. As illustrated in Figure 2, the framework consists of an encoder network *q*_ϕ_(*z*∣*x*) that approximates the posterior distribution of latent variables and a decoder network *p*_θ_(*x*∣*z*) that reconstructs the input data from the latent representation. The model is optimized by maximizing the evidence lower bound (ELBO), which jointly accounts for reconstruction accuracy and a regularization term based on the Kullback-Leibler (KL) divergence between the approximate posterior and the prior distribution, as shown in Equation 1.

(Equation 1) $$\begin{array}{r} \log\;p_{\theta }\left(x\right)\geq L\left(\theta ,\phi ;x\right)\\ =-D_{KL}\left(q_{\phi }\left(z|x\right)∥p_{\theta }\left(z\right)\right)+E_{q_{\phi }\left(z|x\right)}\left[\log\;p_{\theta }\left(x|z\right)\right] \end{array}$$

where *D*_KL_(·) denotes the Kullback-Leibler divergence and *z* represents the latent variable. The ELBO objective balances reconstruction accuracy and distribution regularization, enabling the model to learn meaningful and probabilistically coherent latent representations.

**Figure 2:**
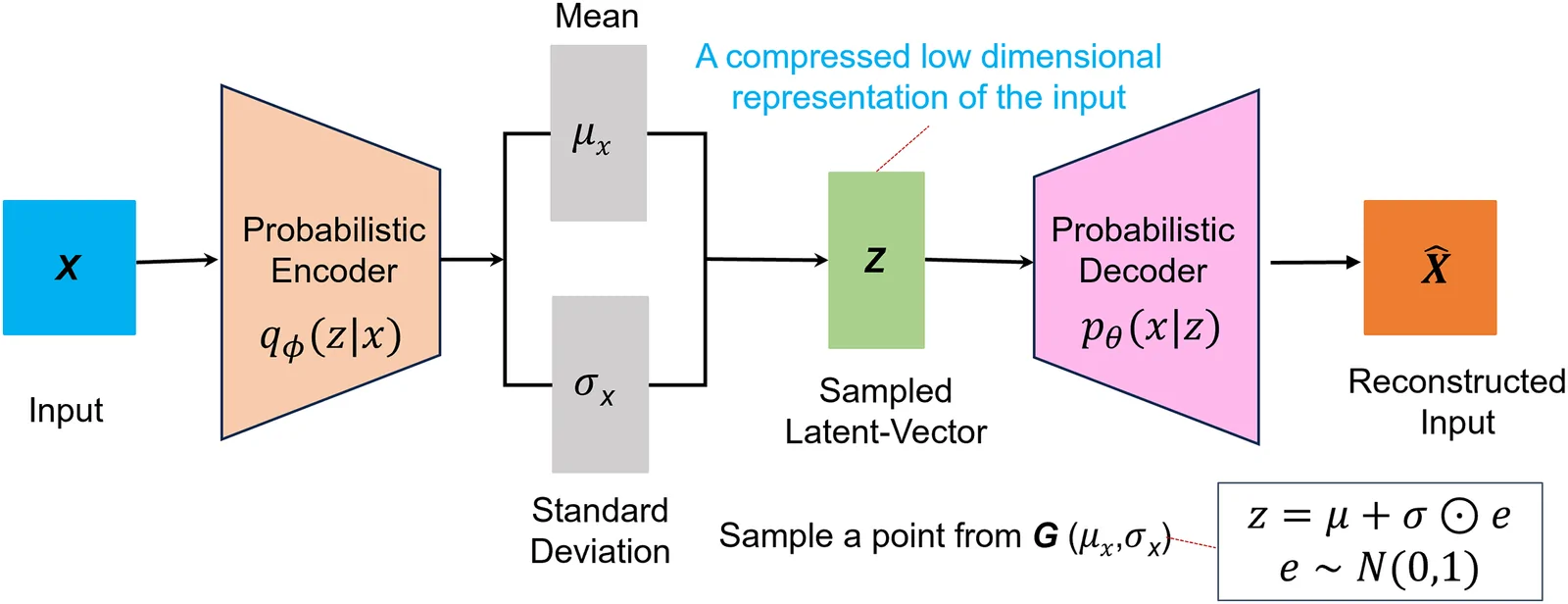
Schematic of the variational autoencoder architecture The input *x* is mapped by the probabilistic encoder *q*_*ϕ*_(*z*∣*x*) to the mean *μ*_*x*_ and standard deviation *σ*_*x*_ of the approximate latent distribution. A latent representation *z* is sampled from this distribution and passed to the probabilistic decoder *p*_*θ*_(*x*∣*z*) to generate the reconstructed input $\hat{x}$. The latent variable *z* provides a compressed low-dimensional representation of the input.

Through this variational objective, VAE learns a structured latent space that encourages smooth interpolation and probabilistic reasoning. When extended to multimodal scenarios, different modalities can be viewed as distinct observations generated from a shared latent variable, enabling the model to capture cross-modal dependencies in a unified probabilistic framework.

Recent studies have explored VAE-based approaches for MER. Yang et al.^28^ leveraged VAE to extract latent affective features for sentiment prediction, demonstrating the effectiveness of probabilistic representation learning. Ong et al.^29^ employed a VAE framework to model latent discourse roles in conversational contexts, enhancing emotion recognition through structured latent variables. Subsequent research^30^^,^^31^^,^^32^^,^^33^ proposed various extensions, including Conditional Variational Autoencoders (CVAEs), which incorporate auxiliary information to guide latent inference and improve modality interaction modeling. In particular, CVAE structures enable conditional generation by allowing available modalities to guide the reconstruction of missing or auxiliary information. Liu et al.^34^ integrated sentiment lexicons into a CVAE-based architecture to jointly learn semantic and affective distributions. Such conditional generative mechanisms are especially promising in missing-modality scenarios, where latent variable sampling can be utilized to infer absent modality representations.

Existing missing-modality multimodal emotion recognition methods can generally be categorized into four groups. The first category is modality translation-based methods, such as MCTN, which recover missing modalities by learning translation mappings between available and missing modalities. Although these methods exploit cross-modal correlations, the accumulated translation errors may negatively affect downstream emotion recognition performance. The second category is reconstruction-based approaches, including MMIN, IF-MMIN, and GCNet, which reconstruct missing modalities from observed modalities and subsequently perform multimodal fusion. However, most of these methods rely on deterministic reconstruction and often overlook the uncertainty inherent in missing-modality scenarios. The third category consists of generative learning methods, such as GMD and LNLN, which model latent feature distributions to improve reconstruction robustness. Nevertheless, the generated representations may still suffer from information loss and unreliable modality estimation under severe modality absence. More recently, Transformer-based approaches, such as NIAT, have demonstrated strong capability in modeling long-range cross-modal dependencies. However, their performance remains highly dependent on the quality and completeness of modality representations.

Despite these advances, existing VAE-based multimodal models typically rely on static fusion schemes and limited contextual modeling, which restrict their ability to handle dynamic and random modality absence. Moreover, many approaches treat temporal dependencies in a simplified manner, potentially weakening cross-modal semantic alignment under incomplete data conditions. These limitations motivate the development of more robust conditional generative frameworks that integrate contextual modeling with probabilistic latent inference—an objective addressed by the proposed RMM-TCVAE.

### Multimodal emotion recognition under missing modalities

Handling missing modalities has become an increasingly important research problem in MER. In practical applications, multimodal inputs are often incomplete due to sensor failure, transmission errors, environmental noise, or user-selective behavior. To enhance robustness under such conditions, existing MER studies can generally be categorized into three methodological paradigms: data-level simulation strategies, generative reconstruction approaches, and joint representation learning frameworks.

The first category focuses on data-level simulation and robustness training. These approaches attempt to improve model generalization by randomly dropping modalities during training or discarding incomplete samples. Li et al.^35^ propose a multi-task pre-training framework that flexibly handles both unimodal and multimodal inputs by selecting lightweight modality experts and shared experts. Xi et al.^36^ introduced a hierarchical knowledge distillation framework that transfers information from complete-modality samples to incomplete inputs. Parthasarathy et al.^37^ simulated real-world missing conditions by randomly suppressing visual inputs at clip or frame levels during training. Although such strategies enhance robustness to certain missing patterns, they often fail to accurately model real-world missing distributions. Random modality ablation may lead to imbalanced modality combinations, inducing distribution bias and degrading MER performance when encountering unseen missing configurations.

The second paradigm relies on generative reconstruction techniques to infer missing modalities. These methods aim to learn the underlying data distribution from complete samples and generate approximated representations for absent modalities. Representative models include autoencoder-based reconstruction,^38^ generative adversarial networks,^39^ and variational autoencoders.^40^ Wen et al.^41^ developed cross-modal multi-view networks with adversarial training to estimate missing views. Feature propagation approaches^42^ reconstruct missing features by leveraging neighboring structural information. While generative reconstruction can improve representation completeness, it may introduce noise accumulation during cross-modal inference and often relies on complex auxiliary architectures. When a substantial portion of training samples contain missing modalities, these models are prone to instability and overfitting.

The third category adopts joint representation learning strategies, exploiting inter-modal correlations to learn robust shared embeddings. Han et al.^43^ proposed a jointly trained architecture consisting of modality-specific encoders and a shared classifier to implicitly fuse audio and visual information for MER. Pham et al.^15^ introduced Multimodal Cyclic Translation Network (MCTN) to learn robust multimodal representations. Zhao et al.^13^ proposed Missing Modality Imagination Network (MMIN), which incorporated cycle consistency learning and residual autoencoders to perform cross-modal imagination in uncertain conditions. Ma et al.^44^ proposed a unified neural representation learning framework designed to extract common representations from heterogeneous training data to support sentiment analysis. However, these methods primarily emphasize common representations while insufficiently preserving modality-specific discriminative cues. Furthermore, many frameworks require tailored architectures for specific missing patterns, limiting adaptability to dynamically changing missing scenarios.

Modality reconstruction has become a prominent direction for handling uncertain missing modalities. Liu et al.^24^ proposed a modality translation framework that reconstructs missing information through cross-modal translation mechanisms, enabling robust sentiment analysis under uncertain modality absence. Building upon this idea, Sun et al.^25^ introduced a similar modality completion strategy that exploits modality similarity to recover missing representations and improve robustness against incomplete multimodal inputs. These studies demonstrate the effectiveness of modality recovery techniques in alleviating information loss caused by missing modalities. Despite these advances, missing-modality MER remains challenging. Severe information loss, noise amplification during reconstruction, and insufficient robustness under high missing rates continue to hinder reliable sentiment prediction. These limitations highlight the necessity of a unified conditional generative framework that simultaneously models shared latent structures, preserves modality-specific information, and adaptively handles diverse missing configurations—motivating the development of the proposed RMM-TCVAE.

Compared with recent missing-modality MER methods that primarily rely on deterministic reconstruction, modality-invariant representation learning, graph completion, noise-aware robustness training, or modality decoupling, RMM-TCVAE provides a unified conditional generative framework for incomplete multimodal emotion recognition. Its novelty lies in jointly integrating Transformer-based cross-modal reconstruction, conditional variational uncertainty modeling, and reliability-aware gated fusion. This design enables the model to infer missing or corrupted modality representations from available cues, explicitly capture reconstruction uncertainty in a shared latent space, and dynamically suppress unreliable reconstructed information during multimodal fusion. Therefore, the proposed framework provides a complementary uncertainty-aware and reliability-adaptive approach to robust multimodal emotion recognition under incomplete-modality conditions.

### Transformer-based cross-modal modeling

Transformer-based architectures have become a dominant paradigm in MER due to their strong capability in modeling long-range dependencies and complex cross-modal interactions. Originally proposed for sequence modeling tasks, the self-attention mechanism enables adaptive weighting of contextual information and has been widely extended to multimodal settings.

In MER, Transformer models are typically employed to capture intra-modal temporal dynamics as well as inter-modal correlations. Multimodal Transformer (MulT)^45^ introduced directional cross-modal attention to align unaligned language, audio, and visual sequences. Subsequent studies have incorporated hierarchical attention mechanisms, multi-head cross-modal attention, and shared Transformer encoders to enhance modality interaction and contextual modeling. Recent advances have further demonstrated the effectiveness of Transformer-based architectures for multimodal emotion analysis. For example, a fine-grained correlation fusion Transformer was proposed to capture subtle cross-modal dependencies and improve emotion classification performance through adaptive interaction modeling.^46^ In addition, hybrid deep learning frameworks integrating contextual representation learning with emotion detection mechanisms have shown promising results in large-scale emotion analysis tasks.^47^ These studies highlight the growing importance of Transformer-based contextual modeling and cross-modal fusion in multimodal affective computing. These approaches significantly improve representation learning by allowing flexible information exchange across modalities, thereby advancing performance in standard complete-modality scenarios.

Despite their effectiveness, most Transformer-based MER models implicitly assume modality completeness during both training and inference. Cross-modal attention mechanisms rely on the availability of all modality representations to compute interaction weights. When one or more modalities are absent, the learned attention distributions may become unreliable or degenerate, resulting in unstable feature aggregation. Although some studies attempt to improve robustness through modality dropout or masking strategies, these approaches primarily serve as regularization techniques rather than principled solutions to incomplete-modality inference.

Furthermore, Transformer architectures are inherently discriminative and do not explicitly model the generative process of multimodal data. While self-attention effectively captures correlations among observed modalities, it lacks the probabilistic mechanism required to infer missing representations in a coherent latent space. Consequently, under random multi-modality missing conditions, purely Transformer-based frameworks may struggle to preserve cross-modal semantic consistency and maintain stable MER performance.

## Results

To evaluate the effectiveness of the proposed RMM-TCVAE under incomplete multimodal conditions, extensive experiments were conducted on three widely used benchmark datasets: CMU-MOSI,^48^ CMU-MOSEI,^49^ and IEMOCAP.^50^ These datasets cover diverse real-world scenarios and have been extensively adopted in MER research. In this section, the proposed RMM-TCVAE framework is systematically evaluated under diverse incomplete multimodal settings. Quantitative comparisons, robustness evaluations, and qualitative visualization analyses are provided to validate its effectiveness and generalization capability.

### Performance comparison under fixed missing rates

Tables 1 and 2 report the performance of RMM-TCVAE and seven representative baseline methods under missing rates ranging from *r* = 0.0 to *r* = 0.5. As expected, the Accuracy (Acc) and Weighted Average F1 scores (WAF) of all methods generally decreased as the missing rate increased, confirming that progressive information loss poses a substantial challenge to the MER task. Nevertheless, RMM-TCVAE achieved the best Acc and WAF results across all evaluated datasets, tasks, and missing-rate settings.

**Table 1:** Comparison of model performance on the MOSI and MOSEI datasets across fixed missing-rate settings

Datasets	Models	Acc (r = 0.0)	WAF (r = 0.0)	Acc (r = 0.1)	WAF (r = 0.1)	Acc (r = 0.2)	WAF (r = 0.2)	Acc (r = 0.3)	WAF (r = 0.3)	Acc (r = 0.4)	WAF (r = 0.4)	Acc (r = 0.5)	WAF (r = 0.5)
CMU-MOSI	MCTN	80.1	81.2	77.5	78.6	75.2	76.4	72.9	74.1	70.8	72.0	68.9	70.2
	MMIN	81.6	82.7	79.2	80.3	76.8	78.1	74.4	75.7	72.2	73.5	70.4	71.8
	IF-MMIN	82.3	83.5	79.9	81.2	77.6	78.9	75.3	76.7	73.1	74.4	71.2	72.6
	GCNet	83.1	84.4	81.6	82.9	79.8	81.0	77.9	79.2	76.2	77.6	74.4	75.8
	NIAT	82.6	83.8	80.8	82.1	78.7	80.1	76.9	78.3	75.1	76.5	73.3	74.7
	GMD	83.4^†^	84.6^†^	81.0	82.4	79.3	80.6	77.6	79.0	75.9	77.2	74.1	75.5
	LNLN	82.9	84.1	81.9^†^	83.2^†^	80.1^†^	81.9^†^	78.4^†^	79.8^†^	77.9^†^	78.3^†^	76.1^†^	76.6^†^
	**Ours**	**84.7**	**86.1**	**83.2**	**84.6**	**82.7**	**83.8**	**80.6**	**82.1**	**80.1**	**81.2**	**78.1**	**79.2**
	Δ_***SOTA***_	↑1.3	↑1.5	↑1.3	↑1.4	↑2.6	↑1.9	↑2.2	↑2.3	↑2.2	↑2.9	↑2.0	↑2.6
CMU-MOSEI	MCTN	78.2	78.0	76.4	76.2	74.0	73.7	71.8	71.4	69.5	69.2	67.3	67.0
	MMIN	79.1	79.5	77.2	77.8	75.0	75.4	72.8	73.3	70.6	71.1	68.5	68.9
	IF-MMIN	80.2	80.8	78.4	78.9	76.5	77.0	74.2	74.8	72.1	72.6	69.8	70.4
	GCNet	81.5	82.2	80.1	80.3	78.8	79.6	77.4	78.1	76.0	76.7	74.4	75.0
	NIAT	80.8	81.5	79.2	79.8	77.6	78.3	76.1	76.7	74.5	75.2	72.9	73.5
	GMD	81.0	81.7	79.5	80.2	78.0	78.7	76.5	77.1	75.0	75.6	73.3	73.9
	LNLN	82.1^†^	82.6^†^	80.9^†^	80.6^†^	79.6^†^	80.6^†^	78.2^†^	78.9^†^	76.7^†^	77.4^†^	75.1^†^	75.8^†^
	**Ours**	**84.8**	**84.7**	**82.3**	**83.1**	**81.9**	**82.6**	**80.2**	**81.2**	**79.6**	**80.1**	**77.6**	**78.4**
	Δ_***SOTA***_	↑2.7	↑2.1	↑1.4	↑2.5	↑2.3	↑2.0	↑2.0	↑2.3	↑2.9	↑2.7	↑2.5	↑2.6

Bold values indicate the best results, while † denotes the second-best results. The row labeled “△_SOTA_” reports the performance difference between RMM-TCVAE and the strongest baseline under the same missing-rate setting, measured in percentage points.

**Table 2:** Performance comparison on the IEMOCAP dataset under fixed missing-rate settings

Datasets	Models	Acc (r = 0.0)	WAF (r = 0.0)	Acc (r = 0.1)	WAF (r = 0.1)	Acc (r = 0.2)	WAF (r = 0.2)	Acc (r = 0.3)	WAF (r = 0.3)	Acc (r = 0.4)	WAF (r = 0.4)	Acc (r = 0.5)	WAF (r = 0.5)
IEMOCAP-4 Class	MCTN	76.9	75.8	75.1	73.4	72.3	72.6	70.8	69.8	68.9	69.5	67.6	66.9
	MMIN	78.5	78.1	76.6	76.4	75.4	74.3	73.1	73.0	71.8	71.1	70.2	69.9
	IF-MMIN	79.3	78.8	77.4	77.3	74.6	75.2	75.5	74.1	71.2	71.9	68.4	70.7
	GCNet	81.2	80.8	79.6	79.1	78.7	78.3	76.9	76.4	75.7	75.3	74.1	73.6
	NIAT	80.4	79.9	79.2	78.6	77.3	77.2	76.4	75.9	74.3	74	73.4	73.0
	GMD	80.8	80.4	79.1	79.3	78.3	77.5	76.3	76.1	75.4	74.9	73.6	73.2
	LNLN	82.3^†^	81.9^†^	80.9^†^	80.5^†^	80.1^†^	79.5^†^	78.0^†^	77.7^†^	77.6^†^	76.9^†^	75.6^†^	75.3^†^
	**Ours**	**83.6**	**84.5**	**82.3**	**83.4**	**82.7**	**82.3**	**80.5**	**79.3**	**80.2**	**79.1**	**77.4**	**77.6**
	Δ_***SOTA***_	↑1.3	↑2.6	↑1.4	↑2.9	↑2.6	↑2.8	↑2.5	↑1.6	↑2.6	↑2.2	↑1.8	↑2.3
IEMOCAP-6 Class–	MCTN	58.5	57.9	57.4	56.3	55.6	55.3	54.9	53.8	53.2	53.4	52.4	51.5
	MMIN	60.3	59.8	59.1	58.9	58.1	57.3	56.6	56.5	55.9	55.0	54.3	54.2
	IF-MMIN	61.1	60.6	60.1	59.4	58.5	58.6	57.8	57.1	56.2	56.4	55.4	54.8
	GCNet	63.2	62.8	62.0	61.9	61.2	60.3	59.5	59.3	58.6	57.8	57.2	56.7
	NIAT	62.4	61.9	61.5	60.7	60.1	59.9	59.2	58.3	57.5	57.5	56.6	55.7
	GMD	63.1	62.5	61.6	61.7	60.7	59.8	59.1	59.1	58.3	57.9	56.8	56.4
	LNLN	64.3^†^	63.9^†^	62.9^†^	62.3^†^	62.2^†^	62.0^†^	60.4^†^	59.8^†^	59.6^†^	59.4^†^	58.1^†^	57.9^†^
	**Ours**	**66.8**	**65.7**	**64.7**	**65.2**	**64.2**	**63.6**	**63.2**	**62.9**	**62.5**	**61.7**	**60.3**	**60.4**
	Δ_***SOTA***_	↑2.5	↑1.8	↑1.8	↑2.9	↑2.0	↑1.6	↑2.8	↑3.1	↑2.9	↑2.3	↑2.2	↑2.5

Bold values indicate the best results, while † denotes the second-best results. The row labeled “Δ_SOTA_” reports the performance difference between RMM-TCVAE and the strongest baseline under the same missing-rate setting, measured in percentage points.

Under complete-modality conditions (*r* = 0.0), RMM-TCVAE already demonstrated competitive representation and fusion capabilities. On CMU-MOSI, it achieved 84.7% Acc and 86.1% WAF, exceeding the strongest baseline by 1.3 and 1.5 percentage points, respectively. On CMU-MOSEI, RMM-TCVAE obtained 84.8% Acc and 84.7% WAF, corresponding to improvements of 2.7 and 2.1 percentage points. Similar advantages were observed on IEMOCAP: the proposed model improved Acc and WAF by 1.3 and 2.6 percentage points in the four-class setting and by 2.5 and 1.8 percentage points in the six-class setting. These results indicate that the proposed architecture remains effective even when all three modalities are available. The performance advantages became more evident under moderate and severe missing conditions. At *r* = 0.3, RMM-TCVAE achieved 80.6% Acc and 82.1% WAF on CMU-MOSI, outperforming the strongest baseline by 2.2 and 2.3 percentage points. On CMU-MOSEI, the corresponding improvements were 2.0 and 2.3 percentage points. Particularly notable results were obtained on the IEMOCAP six-class task, where RMM-TCVAE achieved 63.2% Acc and 62.9% WAF, exceeding the strongest baseline by 2.9 and 3.1 percentage points, respectively. The 3.1-point WAF improvement represents the largest gain observed across the fixed missing-rate experiments. At the highest missing rate (*r* = 0.5), RMM-TCVAE continued to maintain a clear advantage over all competing methods. Specifically, it achieved 78.1% Acc and 79.2% WAF on CMU-MOSI, outperforming the strongest baseline by 2.0 and 2.6 percentage points. On CMU-MOSEI, the proposed model obtained 77.6% Acc and 78.4% WAF, representing gains of 2.5 and 2.6 percentage points. For the IEMOCAP four-class task, RMM-TCVAE surpassed the strongest baseline by 1.8 points in Acc and 2.3 points in WAF. In the more challenging six-class task, it achieved 60.3% Acc and 60.4% WAF, exceeding the strongest competing method by 2.2 and 2.5 percentage points.

Overall, although increasing missing rates degraded the performance of all models, RMM-TCVAE consistently retained the leading position across the four evaluation tasks. Figure 3 illustrates the performance trends of different models on IEMOCAP across varying missing rates. It can be observed that all models exhibited decreasing performance as the missing rate increases. However, RMM-TCVAE consistently outperformed competing methods across all missing-rate settings. In particular, the performance advantage remained clear and was particularly evident in several higher missing-rate settings, indicating that the proposed model is more robust in handling severe incomplete-modality conditions.

**Figure 3:**
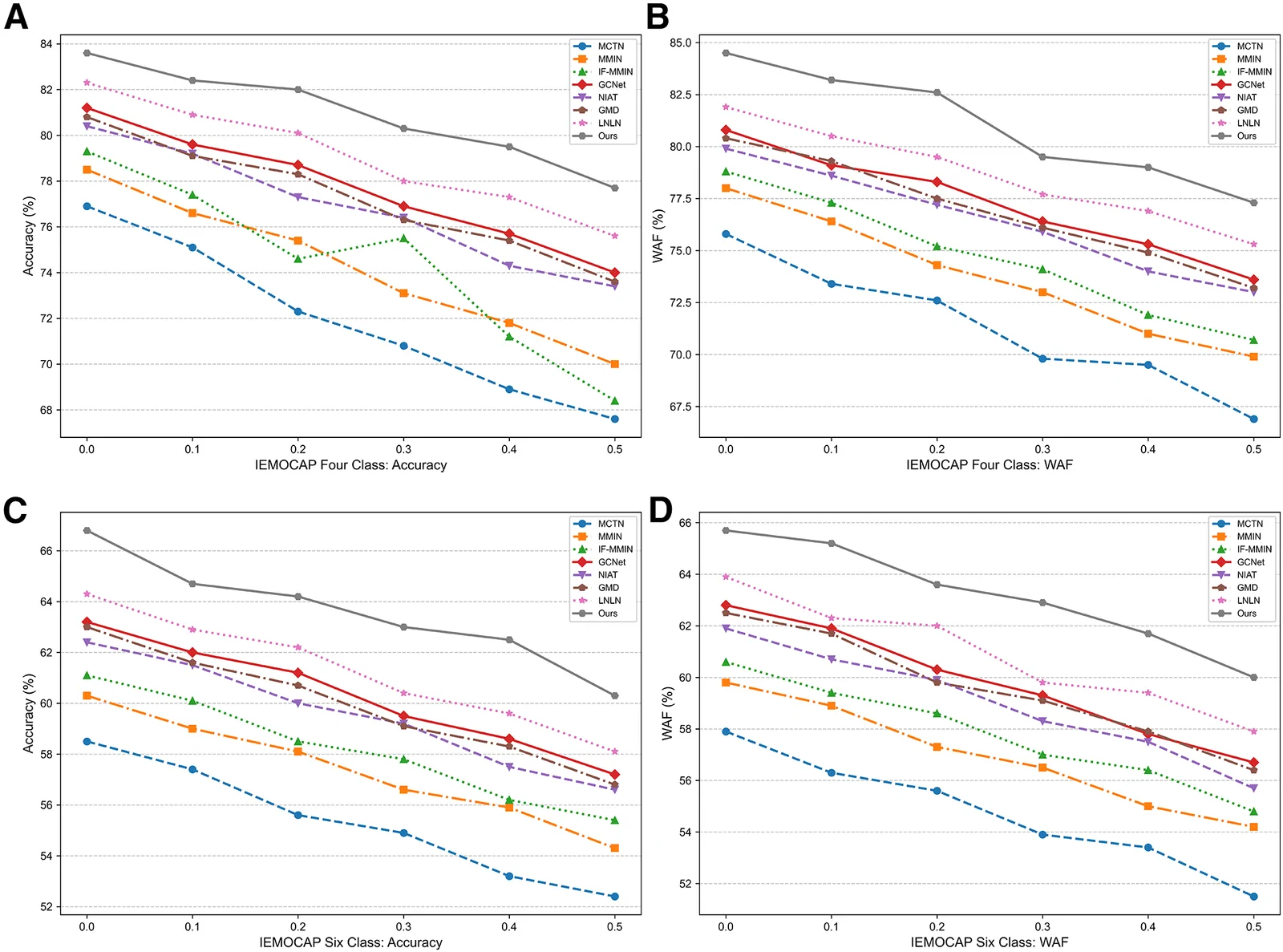
Performance comparison on IEMOCAP across increasing missing rates Performance of MCTN, MMIN, IF-MMIN, GCNet, NIAT, GMD, LNLN, and RMM-TCVAE is compared under missing rates from *r* = 0.0 to *r* = 0.5. (A) Accuracy (Acc) for the four-class task. (B) Weighted average F1-score (WAF) for the four-class task. (C) Acc for the six-class task. (D) WAF for the six-class task. Higher values indicate better recognition performance. Results are averaged across 5-fold speaker-independent cross-validation and three random seeds.

### Performance under different missing-modality patterns

To further evaluate robustness, we examine scenarios where one or more modalities are entirely removed. Tables 3 and 4 report the results under single-modality, dual-modality, and complete-modality configurations. As observed from the experimental results, the absence of textual modality led to the most significant performance drop, confirming its dominant role in semantic polarity modeling. For example, under single-modality settings, text-only input achieved substantially higher performance than audio-only and visual-only inputs on CMU-MOSI, with Acc/WAF scores of 78.9%/79.6%, compared with 63.4%/62.9% for audio-only and 60.8%/60.2% for visual-only. A similar trend was observed on CMU-MOSEI, where text-only input reached 75.3%/75.9%, outperforming audio-only and visual-only inputs by a clear margin. On IEMOCAP, text-only input also achieved the best single-modality performance, with Acc/WAF scores of 69.7%/69.1% in the four-class setting and 53.9%/53.2% in the six-class setting.

**Table 3:** Performance of the proposed RMM-TCVAE on the MOSI and MOSEI datasets under different missing-modality settings

Datasets	Input	Acc (r = 0.0)	WAF (r = 0.0)	Acc (r = 0.1)	WAF (r = 0.1)	Acc (r = 0.2)	WAF (r = 0.2)	Acc (r = 0.3)	WAF (r = 0.3)	Acc (r = 0.4)	WAF (r = 0.4)	Acc (r = 0.5)	WAF (r = 0.5)
CMU-MOSI	{a}	63.4	62.9	–	–	–	–	–	–	–	–	–	–
	{v}	60.8	60.2	–	–	–	–	–	–	–	–	–	–
	{t}	78.9	79.6	–	–	–	–	–	–	–	–	–	–
	{a, v}	69.8	69.3	65.7	67.1	62.4	64.5	61.6	62.8	60.9	60.4	59.5	59.8
	{a, t}	82.1	82.9	74.1	74.6	73.5	73.2	70.1	72.3	70.3	68.9	65.8	68.1
	{v, t}	74.3	73.9	70.2	71.5	69.6	71	68.3	69.7	68.7	67.8	64.8	66.7
	**{a, v, t}**	**84.7**	**86.1**	**83.2**	**84.6**	**82.7**	**83.8**	**80.6**	**82.1**	**80.1**	**81.2**	**78.1**	**79.2**
CMU-MOSEI	{a}	61.2	60.8	–	–	–	–	–	–	–	–	–	–
	{v}	59.8	59.2	–	–	–	–	–	–	–	–	–	–
	{t}	75.3	75.9	–	–	–	–	–	–	–	–	–	–
	{a, v}	67.5	67.1	66	65.6	65.2	64.8	63.4	63.2	62.5	61.5	60.6	60.5
	{a, t}	80.2	80.8	79.3	79.5	78.1	78.6	76.5	77.5	76	76.3	74.1	75.2
	{v, t}	72.6	72.2	71	70.6	70.3	69.8	68.9	68.4	67.8	67.1	66.2	65.6
	**{a, v, t}**	**84.8**	**84.7**	**82.3**	**83.1**	**81.9**	**82.6**	**80.2**	**81.2**	**79.6**	**80.1**	**77.6**	**78.4**

The input {a}, {v}, and {t} represent audio, visual, and text modalities, respectively, and combinations such as {a, t} indicate the available input modalities. “–” indicates that the corresponding missing-rate setting is not applicable for single-modality input conditions. Bold values indicate the best results under each dataset and missing-rate setting.

**Table 4:** Performance of the proposed RMM-TCVAE on the IEMOCAP under different missing-modality settings

Datasets	Input	Acc (r = 0.0)	WAF (r = 0.0)	Acc (r = 0.1)	WAF (r = 0.1)	Acc (r = 0.2)	WAF (r = 0.2)	Acc (r = 0.3)	WAF (r = 0.3)	Acc (r = 0.4)	WAF (r = 0.4)	Acc (r = 0.5)	WAF (r = 0.5)
IEMOCAP-4 Class	{a}	59.1	58.4	–	–	–	–	–	–	–	–	–	–
	{v}	55.8	55.0	–	–	–	–	–	–	–	–	–	–
	{t}	69.7	69.1	–	–	–	–	–	–	–	–	–	–
	{a, v}	62.6	61.9	59.5	60.5	55.4	56.6	51.8	53.2	46.2	45	41.9	42.8
	{a, t}	75.1	74.6	71.1	72.6	67.6	65.4	62.8	61.4	56.8	55.3	52	51
	{v, t}	71.2	70.6	68.9	67	66.1	66.5	61.7	60.6	56.3	57.5	50.6	48.7
	**{a, v, t}**	**83.6**	**84.5**	**82.3**	**83.4**	**82.7**	**82.3**	**80.5**	**79.3**	**80.2**	**79.1**	**77.4**	**77.6**
IEMOCAP-6 Class	{a}	45.6	44.9	–	–	–	–	–	–	–	–	–	–
	{v}	42.3	41.6	–	–	–	–	–	–	–	–	–	–
	{t}	53.9	53.2	–	–	–	–	–	–	–	–	–	–
	{a, v}	48	47.3	47.6	46.3	46	45.5	45.1	44.5	44	43.2	42.7	41.9
	{a, t}	60.1	59.6	59.4	58.7	58.2	58	57.6	56.6	56.3	55.3	55	54
	{v, t}	56.7	56.2	56.3	55.4	55.4	54.4	54.3	53.3	53.2	52.1	51.8	50.8
	**{a, v, t}**	**66.8**	**65.7**	**64.7**	**65.2**	**64.2**	**63.6**	**63.2**	**62.9**	**62.5**	**61.7**	**60.3**	**60.4**

The input {a}, {v}, and {t} represent audio, visual, and text modalities, respectively, and combinations such as {a, t} indicate the available input modalities. “–” indicates that the corresponding missing-rate setting is not applicable for single-modality input conditions. Bold values indicate the best results under each dataset and missing-rate setting.

The dual-modality results further show that performance varies substantially across different missing patterns. When the visual modality was removed, the audio-text setting achieved 82.1% Acc and 82.9% WAF on CMU-MOSI, showing only a moderate decrease from the complete-modality setting of 84.7%/86.1%. In contrast, removing the textual modality resulted in much lower performance under the audiovisual setting, with 69.8% Acc and 69.3% WAF. Similar patterns were observed on CMU-MOSEI, where the audio-text setting achieved 80.2%/80.8%, compared with 84.8%/84.7% under complete-modality input and 67.5%/67.1% under audiovisual input. These results indicate that removing the visual modality caused comparatively smaller degradation than removing the textual modality, suggesting that textual information provides the most critical semantic cues, whereas visual features may be more sensitive to noise and frame-level inconsistencies in these datasets. Moreover, dual-modality inputs generally outperformed single-modality inputs, demonstrating the complementary contribution of multimodal cues. Among dual-modality inputs, the audio-text combination yielded stronger performance than visual-text and audiovisual combinations. For instance, on CMU-MOSI, the audio-text setting achieved 82.1%/82.9%, outperforming visual-text (74.3%/73.9%) and audiovisual (69.8%/69.3%) settings. Similar results were obtained on CMU-MOSEI, where audio-text reached 80.2%/80.8%, compared with 72.6%/72.2% for visual-text and 67.5%/67.1% for audiovisual input. This pattern also appeared on IEMOCAP, where audio-text achieved 75.1%/74.6% in the four-class setting and 60.1%/59.6% in the six-class setting.

In addition, within the dual-modality and complete-modality configurations where missing rates are applicable, performance generally decreased as the missing rate increased. For example, on CMU-MOSI, the complete-modality setting declined from 84.7%/86.1% at *r* = 0.0 to 78.1%/79.2% at *r* = 0.5, while the audio-text setting {a, t} decreased from 82.1%/82.9% to 65.8%/68.1%. A similar degradation trend was also observed on CMU-MOSEI and IEMOCAP, indicating that higher missing rates consistently increase the difficulty of robust multimodal representation learning.

### Ablation study

To investigate the contribution of each key component in the proposed RMM-TCVAE framework, we conducted ablation experiments on the IEMOCAP dataset by systematically removing three critical components: the TRN, the GMFN, and the reconstruction loss term $L_{\text{rec}}$. The results are summarized in Table 5, confirming that each component contributes to performance improvements. First, removing the Transformer-based reconstruction network (-w/o TRN) resulted in the most substantial performance degradation across all missing-rate settings. At the highest missing rate, removing TRN caused an approximately 6.6 percentage-point performance drop compared with the full model. This demonstrated that generative reconstruction played a crucial role in mitigating representation collapse under severe modality absence. Second, eliminating the Gated Multimodal Fusion Network (-w/o GMFN) also led to a noticeable performance decline, indicating the importance of gated attention mechanisms in capturing complementary cross-modal interactions. Third, removing the reconstruction loss (-w/o $L_{\text{rec}}$) caused moderate degradation. Although the model could still perform reasonably well, the absence of the reconstruction objective weakened the fidelity and semantic consistency of reconstructed modality representations. Moreover, it is worth noting that the performance gap between the full model and its ablated variants became more pronounced as the missing rate increased. This trend further confirmed that the joint design of conditional reconstruction and adaptive fusion significantly enhanced robustness under incomplete multimodal scenarios. Overall, the ablation results validated the positive contribution of each module, and their integration enabled effective learning of both modality-specific and modality-shared representations, thereby reducing cross-modal distribution gaps.

**Table 5:** Ablation experiments on the IEMOCAP dataset

Datasets	Modules	Acc (r = 0.0)	WAF (r = 0.0)	Acc (r = 0.1)	WAF (r = 0.1)	Acc (r = 0.2)	WAF (r = 0.2)	Acc (r = 0.3)	WAF (r = 0.3)	Acc (r = 0.4)	WAF (r = 0.4)	Acc (r = 0.5)	WAF (r = 0.5)
IEMOCAP-4 Class	-w/o TRN	79.8	79.3	78.4	78.5	75.3	76.4	74.2	73.1	72.8	72.9	70.1	71.0
	-w/o GMFN	81.7	81.2	80.2	80.1	79.3	78.3	77.2	77.4	76.4	75.2	74.8	74.3
	-w/o $L_{\text{rec}}$	82.4	81.9	81.3	81.2	80.5	79.4	78.7	78.2	77.9	76.8	76.0	75.4
	**Ours**	**83.6**	**84.5**	**82.3**	**83.4**	**82.7**	**82.3**	**80.5**	**79.3**	**80.2**	**79.1**	**77.4**	**77.6**
IEMOCAP-6 Class	-w/o TRN	61.4	60.9	60.3	59.2	58.5	58.5	57.8	56.7	55.8	55.7	54.4	54.3
	-w/o GMFN	63.2	62.7	61.8	61.7	60.9	60.1	59.2	59.3	58.6	57.4	56.9	56.6
	-w/o $L_{\text{rec}}$	64.6	64.1	63.5	63.4	63.0	62.1	61.4	61.3	60.7	60.2	59.4	58.9
	**Ours**	**66.8**	**65.7**	**64.7**	**65.2**	**64.2**	**63.6**	**63.2**	**62.9**	**62.5**	**61.7**	**60.3**	**60.4**

“-w/o” denotes the removal of the corresponding module or loss term. Bold values indicate the best results under each task and missing-rate setting.

### Convergence analysis

To further examine the training stability and convergence of the proposed RMM-TCVAE framework, we analyzed the optimization process on the CMU-MOSEI and IEMOCAP datasets under a fixed missing rate of 0.2. This setting represented a moderate modality-incomplete scenario and served as a representative case for convergence examination. Figure 4 illustrates the evolution of the training loss and validation loss across epochs. The overall objective consisted of the classification loss, the reconstruction loss $L_{\text{rec}}$, and the KL divergence regularization term. As observed from the convergence curves, although minor oscillations appeared during early training stages due to stochastic optimization and modality masking randomness, both the training and validation losses exhibited a clear downward trend. The model gradually stabilized and converged after approximately 40–50 epochs. Notably, the validation loss achieved its optimal performance within this interval, indicating that the model achieved stable optimization without obvious training instability.

**Figure 4:**
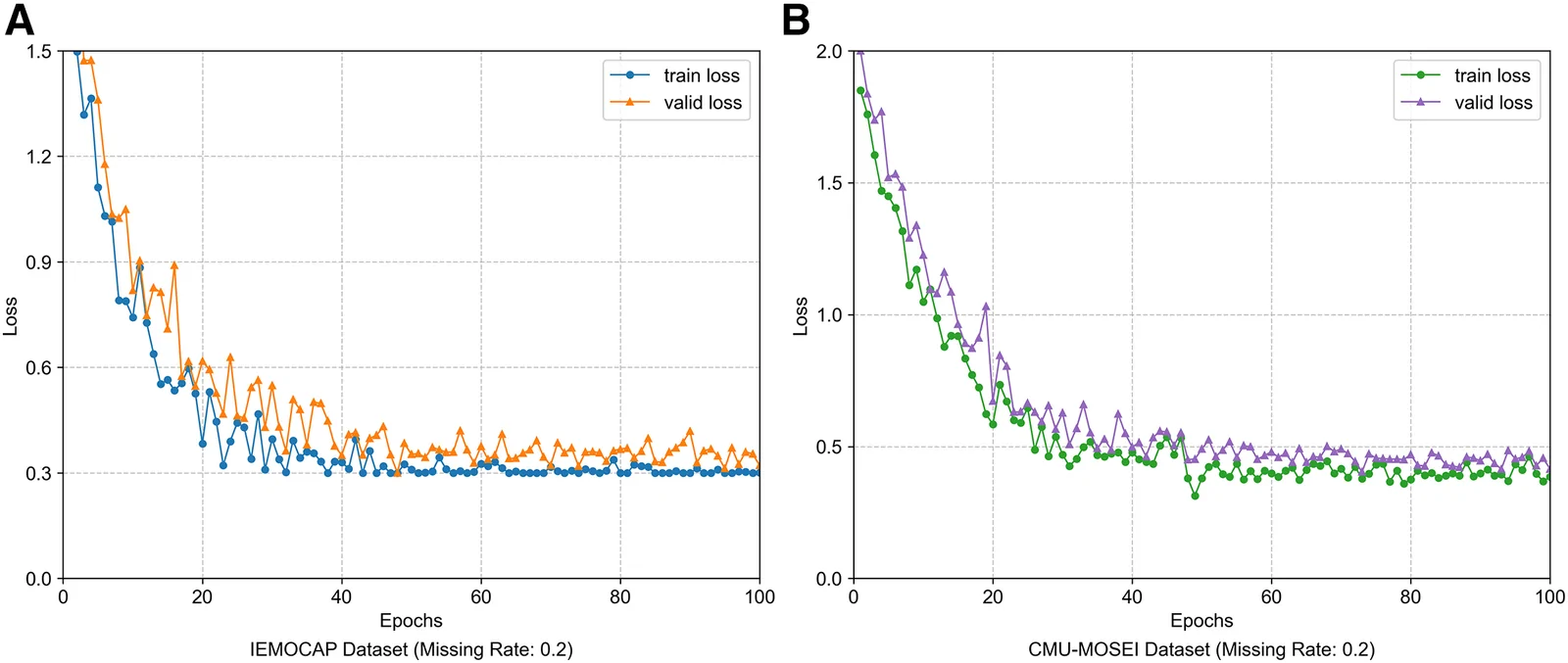
Training and validation loss convergence of RMM-TCVAE at a missing rate of *r* = 0.2 Among them, (A) shows the convergence curves on the IEMOCAP (four-class) dataset, and (B) shows the convergence curves on the CMU-MOSEI dataset. The horizontal axis denotes training epochs, and the vertical axis denotes the overall optimization loss, which combines classification, reconstruction, and Kullback-Leibler divergence terms.

### Embedding space visualization analysis

To qualitatively assess the learned representations, we visualized the latent embeddings on the IEMOCAP (four-class) test set under a missing rate of 0.2. Figure 5 presents the t-SNE projections, where red, blue, green, and purple correspond to happiness, sadness, neutral, and anger, respectively. As observed, the proposed method produced highly structured and well-separated clusters in the latent space. Compared with conventional multimodal learning approaches, which often exhibit substantial overlap among emotional categories under missing-modality conditions, the proposed model achieved clearer inter-class boundaries and more compact intra-class distributions. This suggests that the model effectively captured modality-invariant yet emotion-discriminative features, thereby mitigating the adverse impact of incomplete inputs on representation quality.

**Figure 5:**
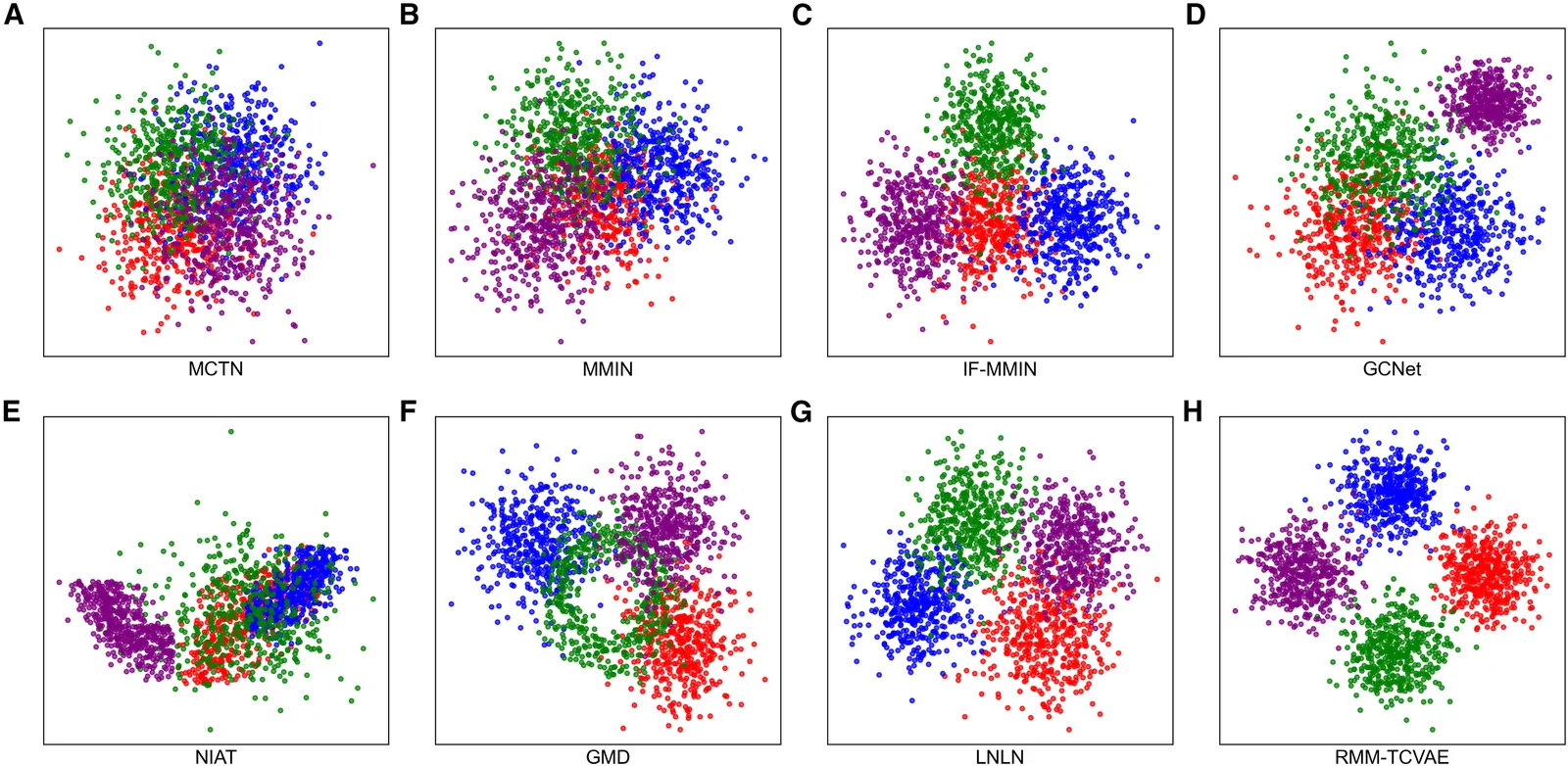
Comparative t-SNE visualization of the latent representations on the IEMOCAP four-class dataset with a missing rate of *r* = 0.2 Two-dimensional t-SNE projections of latent representations are shown for (A) MCTN, (B) MMIN, (C) IF-MMIN, (D) GCNet, (E) NIAT, (F) GMD, (G) LNLN, and (H) RMM-TCVAE. Each point represents one test utterance. Red, blue, green, and purple denote happiness, sadness, neutral, and anger, respectively. The same color mapping is used across all panels for comparison.

To further examine the training dynamics, we analyzed the evolution of latent representations across different training stages under the same missing rate. Figure 6 presents t-SNE visualizations at the 10th, 20th, 30th, and 40th epochs, using a consistent color scheme as in Figure 5. At earlier stages (e.g., epoch 10), the representations of different emotional classes are relatively entangled, indicating insufficient discrimination in the latent space. As training proceeds, the clusters gradually become more distinguishable, with increasing inter-class margins and improved intra-class cohesion. Notably, the feature distributions did not collapse into overly dense regions, but instead maintained an appropriate level of dispersion, which reflects a balance between compactness and diversity. This behavior implies that the proposed method avoids representation collapse while progressively enhancing class separability. Since t-SNE provides qualitative and projection-dependent evidence, these visualization results are interpreted together with the quantitative missing-rate comparisons and ablation results.

**Figure 6:**
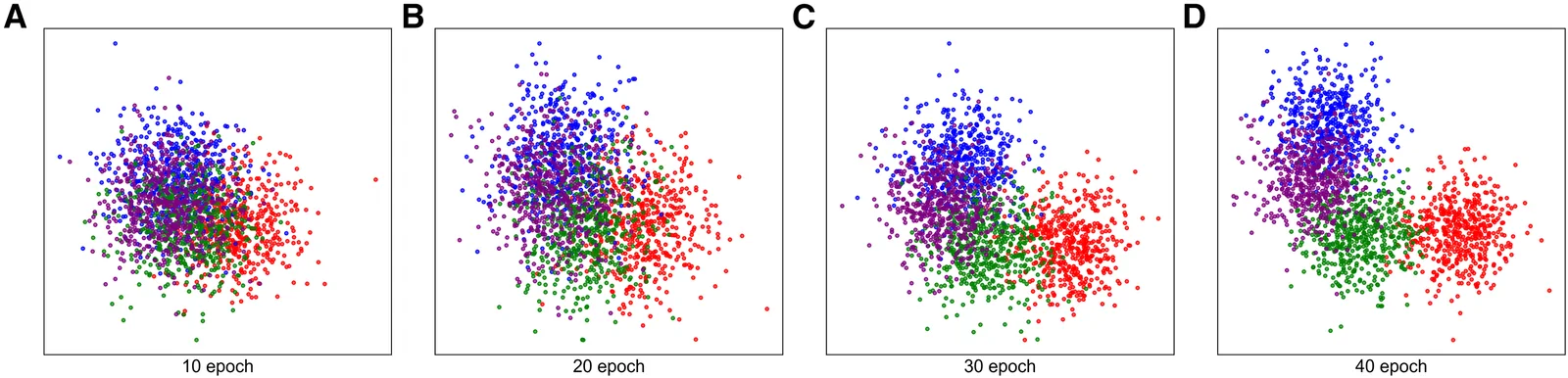
Evolution of latent representations during RMM-TCVAE training on the IEMOCAP four-class test set at *r* = 0.2 Two-dimensional t-SNE projections of latent representations are shown at different training stages under a missing rate of *r* = 0.2: (A) epoch 10, (B) epoch 20, (C) epoch 30, and (D) epoch 40. Each point represents one test utterance. Red, blue, green, and purple denote happiness, sadness, neutral, and anger, respectively. The same color mapping is used across all panels.

### Prediction performance analysis based on confusion matrices

To evaluate the prediction capability of different models, normalized confusion matrices are presented based on the experimental results on the IEMOCAP test set. As shown in Figure 7, the matrices illustrate the classification performance of several baseline models and the proposed model. From Figure 7, it can be observed that the proposed model achieved strong performance in recognizing the “Sad” and “Excited” emotion categories. However, distinguishing between “Happy” and “Neutral” remained challenging for the model. For instance, as shown in the confusion matrix of the proposed method (Figure 7F), when the true label is “Neutral”, approximately 16.1% of the samples are misclassified as “Happy”, and about 11.8% are predicted as “Excited”. This indicated that these emotion categories shared similar characteristics in the feature space, making them more difficult to distinguish. Overall, the “Sad” class showed the highest recognition accuracy among the six emotion categories, suggesting that it contains more distinctive features that could be effectively captured by the model. In contrast, “Happy” samples are more likely to be confused with other emotional categories, resulting in a relatively larger prediction error. Compared with other baseline models, the proposed approach improved the recognition performance for most emotion categories. Although the accuracy for the “Excited” class showed a slight decrease in some cases, the overall classification performance was enhanced, particularly for the “Sad” category. These results demonstrated that the proposed method provided a more robust representation under missing-modality conditions, thereby improving the model’s ability to recognize emotions in multimodal scenarios.

**Figure 7:**
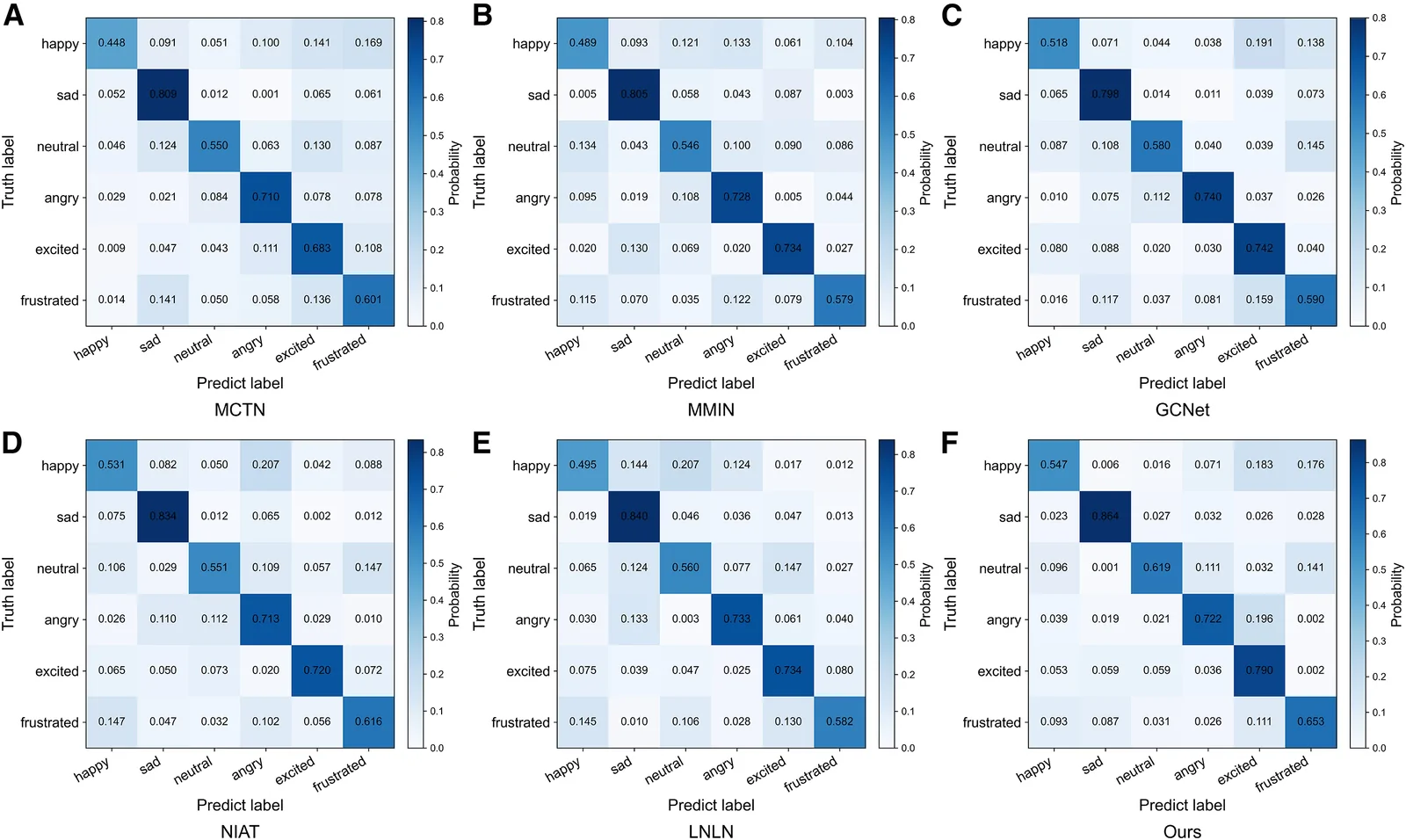
Comparison of normalized confusion matrices on the IEMOCAP six-class task Normalized confusion matrices are shown for (A) MCTN, (B) MMIN, (C) GCNet, (D) NIAT, (E) LNLN, and (F) RMM-TCVAE. Rows represent true emotion labels and columns represent predicted labels for the six classes: happy, sad, neutral, angry, excited, and frustrated. Each cell reports the row-normalized predicted probability for the corresponding true-predicted label pair. Color intensity represents the predicted probability, with darker diagonal cells indicating higher classification accuracy for the corresponding emotion class.

### Model complexity analysis

To evaluate the computational complexity of different missing-modality multimodal emotion recognition methods, we calculated the number of trainable parameters (Parameters) and floating-point operations (FLOPs) for several representative models. As shown in Table 6, we compared the proposed RMM-TCVAE with MCTN, MMIN, IF-MMIN, GCNet, NIAT, GMD, and LNLN. To ensure a fair comparison, all complexity statistics were computed under a unified experimental setting on the CMU-MOSEI dataset. The pretrained BERT text encoder was excluded from the complexity calculation because it serves as a common feature extractor rather than a task-specific component of the multimodal learning framework. Therefore, the reported Parameters and FLOPs mainly reflect the computational cost of the downstream multimodal reconstruction, representation learning, fusion, and classification modules. As shown in Table 6, the proposed RMM-TCVAE achieves the best overall recognition performance among all compared methods. Specifically, RMM-TCVAE attains the highest Accuracy (84.8%) and WAF (84.7%), outperforming the strongest baseline LNLN by 2.7 and 2.1 percentage points, respectively. Notably, this performance gain is achieved with only 2.4M trainable parameters, which is the second-smallest model among all methods and only slightly larger than MMIN (2.3M). In contrast, several recent approaches, such as GCNet (4.9M parameters) and NIAT (8.1M parameters), require substantially larger model capacities but achieve lower recognition performance. In terms of computational cost, RMM-TCVAE requires 3.9G FLOPs, which is considerably lower than MCTN (4.5G), GMD (4.4G), LNLN (4.7G), and especially NIAT (9.2G), while remaining comparable to GCNet (3.8G). Although MMIN and IF-MMIN exhibit lower FLOPs, their Acc and WAF scores are significantly inferior to those of RMM-TCVAE. These results indicate that the proposed framework achieves a favorable performance-complexity trade-off.

**Table 6:** Comparison of model complexity and performance on the CMU-MOSEI dataset

Methods	Parameters (M)	FLOPs (G)	Acc (%)	WAF (%)
MCTN	3.2	4.5	78.2	78.0
MMIN	**2.3**	**1.6**	79.1	79.5
IF-MMIN	3.1	2.2	80.2	80.8
GCNet	4.9	3.8	81.5	82.2
NIAT	8.1	9.2	80.8	81.5
GMD	2.9	4.4	81.0	81.7
LNLN	3.3	4.7	82.1	82.6
**Ours**	2.4	3.9	**84.8**	**84.7**

Performance values are reported under the fixed missing-rate setting with r = 0.0. Bold values indicate the best value in each column, where lower parameters/FLOPs and higher Acc/WAF are preferred.

To further evaluate the practical efficiency of RMM-TCVAE, we additionally measured its runtime and memory consumption on the CMU-MOSEI dataset. As shown in Table 7, RMM-TCVAE requires 51.3 s per training epoch and consumes 3.92 GB of peak GPU memory during training. During inference, the model requires an average of 2.8 ms per test sample under a batch size of 32 and consumes 0.54 GB of peak GPU memory. Although the model introduces additional memory consumption during training, it remains within an acceptable range for modern GPU-based experimental settings, and the low inference time suggests that the model can be efficiently applied to incomplete-modality emotion recognition tasks. Comprehensive analysis indicates that the current memory and time overhead mainly stems from the conditional reconstruction and reliability-aware fusion processes designed to handle uncertain missing-modality data. For reference, the dataset statistics and standard evaluation protocols are summarized in Table 8, and the experimental hyperparameter configurations are provided in Table 9.

**Table 7:** Runtime and memory profile of RMM-TCVAE on the CMU-MOSEI dataset

Model	Training time/epoch	Training memory	Inference time/sample	Inference memory	FLOPs
Ours	51.3s	3.92 GB	2.8 ms	0.54 GB	3.9G

Runtime and memory profiling were conducted on CMU-MOSEI using the same batch size of 32 as reported in Table 9.

**Table 8:** Dataset statistics and standard evaluation protocols

Dataset	Total utterances	Total videos/Sessions	Training set	Validation set	Test set	Evaluation Protocol
CMU-MOSI	2,199	93 videos	1,284	229	686	official split
CMU-MOSEI	22,856	3,225 videos	16,326	1,871	4,659	official split
IEMOCAP (4-class)	5,531	5 sessions	–	–	–	5-fold CV
IEMOCAP (6-class)	7,433	5 sessions	–	–	–	5-fold CV

For IEMOCAP, “–” indicates that no fixed training, validation, or test split is used.

**Table 9:** Configuration of parameters used in our experiments on different datasets

Parameters	CMU-MOSI	CMU-MOSEI	IEMOCAP
Batch size	32	32	64
Epoch number	200	200	200
Optimizer	AdamW	AdamW	AdamW
Initial learning rate	1e−4	1e−4	1e−4
Learning rate_BERT	5e−5	5e−5	5e−5
Dropout rate	0.1	0.1	0.1
Missing rate	[0.1, 0.5]	[0.1, 0.5]	[0.1, 0.5]
Transformer encoder	*n* = 4, h = 2, e = 128	*n* = 4, h = 2, e = 128	*n* = 4, h = 2, e = 128
Warm up	✓	✓	✓
Early stop	✓	✓	✓
Cosine Annealing	✓	✓	✓
Seed	111,112,113	111,112,113	111,112,113
*λ*_1_	0.1	0.1	0.1
*λ*_2_	0.01	0.01	0.01

In the Transformer encoder setting, n denotes the number of Transformer layers, h denotes the number of attention heads, and e denotes the hidden embedding dimension.

## Discussion

This study proposes RMM-TCVAE, a Transformer-based conditional variational autoencoder framework for MER under missing-modality conditions. By integrating Transformer-based reconstruction, conditional variational latent modeling, and reliability-aware gated fusion, the proposed framework aims to recover missing-modality representations while reducing the adverse influence of uncertain or noisy reconstructed features. The missing-modality configurations considered in this study are summarized in Figure 8, while the overall RMM-TCVAE architecture and the gated multimodal fusion mechanism are illustrated in Figures 9 and 10, respectively. The complete training workflow is summarized in Algorithm 1. Experiments on CMU-MOSI, CMU-MOSEI, and IEMOCAP demonstrate that RMM-TCVAE achieves consistent improvements across different missing-modality settings, supporting its effectiveness for robust MER.

**Figure 8:**
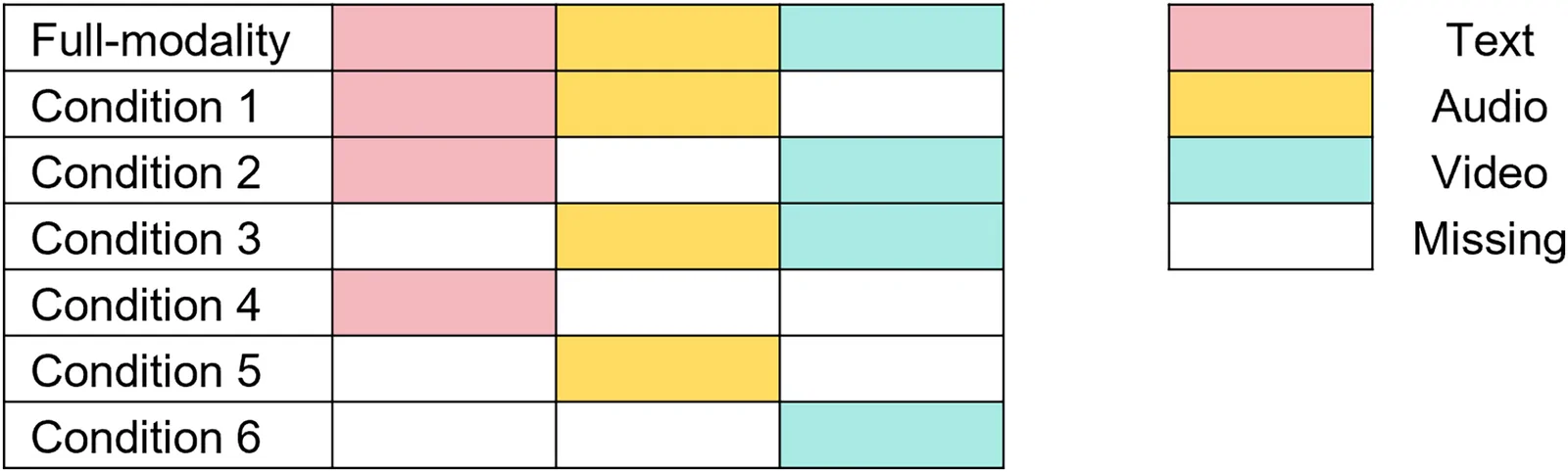
Possible missing-modality configurations in multimodal data The full-modality setting and six possible modality-level missing configurations involving text, audio, and visual modalities are shown. Pink, yellow, and cyan blocks represent text, audio, and visual modalities, respectively, whereas blank positions indicate unavailable modalities. The configurations include three dual-modality settings with one modality unavailable and three single-modality settings with two modalities unavailable.

**Figure 9:**
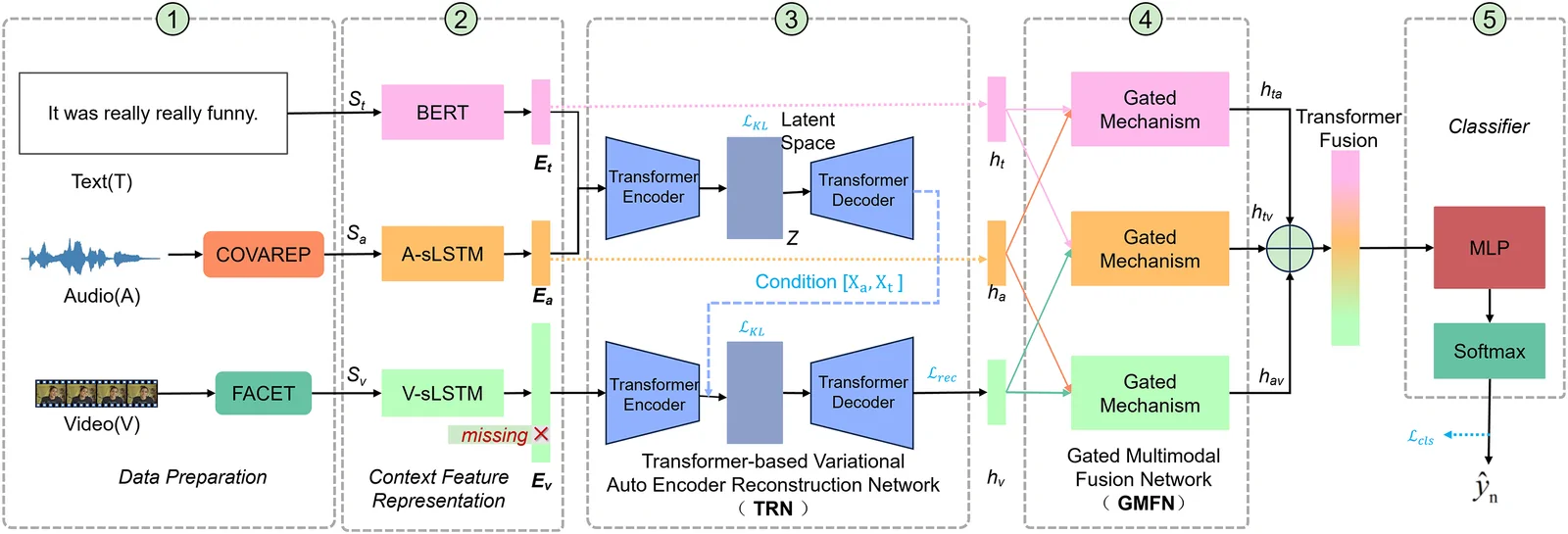
Overall architecture of RMM-TCVAE for multimodal emotion recognition under missing modalities The overall architecture consists of five main components: (1) Data preprocessing, (2) Contextual feature representation, (3) Transformer-based conditional variational Reconstruction Network (TRN), (4) Gated Multimodal Fusion Network (GMFN), and (5) Sentiment classifier. For clarity, the framework is illustrated using the visual modality missing scenario as an example. The observed text and audio representations condition the reconstruction of the missing visual modality in the TRN, after which the observed and reconstructed representations are adaptively integrated by the GMFN and passed to the MLP. *L*_*KL*_, *L*_*rec*_, and *L*_*cls*_ denote the Kullback-Leibler divergence, reconstruction, and classification losses, respectively. Pink, orange, and green pathways represent text, audio, and visual modalities, respectively.

**Figure 10:**
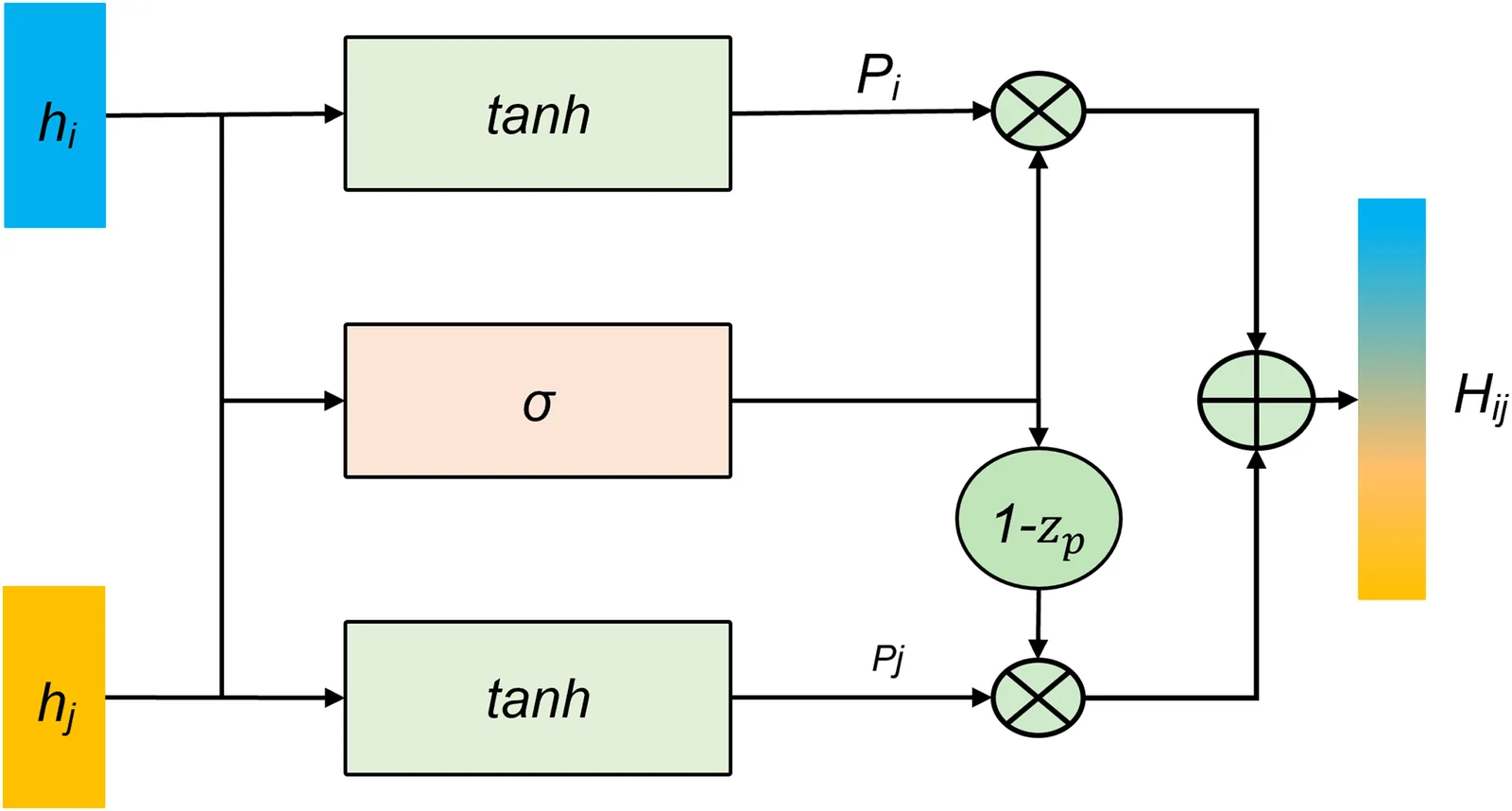
Architecture of gated multimodal fusion network (GMFN) for reliability-aware fusion of observed and reconstructed modality representations Two modality representations, *h*_*i*_ and *h*_*j*_, are first transformed through nonlinear mappings to obtain modality-specific representations. A sigmoid gate *z*_*p*_ adaptively controls their relative contributions, while the complementary weights *z*_*p*_ and 1-*z*_*p*_ are applied through element-wise multiplication. The weighted representations are then combined to obtain the pairwise fused representation *H*_*ij*_. The same gated interaction is applied to the audio-visual, audio-text, and visual-text modality pairs to generate *H*_*av*_, *H*_*at*_, and *H*_*vt*_, respectively, before subsequent multimodal fusion.

Algorithm 1 **Pseudocode for the proposed RMM-TCVAE**

**Input**: Multimodal dataset $D={\left\{\left(X^{\left(i\right)},y^{\left(i\right)}\right)\right\}}_{i=1}^{N}$, X = [X_t_,X_a_,X_v_]; missing rate r; hyperparameters *λ*_1_,*λ*_2_; epoch

1: Initialize all model parameters Θ

2: **for** i: [1, N] **do** Data Processing to extract baseline features

3: **while** epoch >0 **do**

4:  Generate a modality mask matrix *M*∈{0,1}^*B*×3^ according to the missing rate *r*

5:  Apply *M* to obtain incomplete multimodal samples $X'=[X{'}_{a},\;X{'}_{v},\;X{'}_{t}]$

6:  Context Feature Representation via: $E_{m}=sLSTM\;\left(S_{m};\;\theta_{m}^{lstm}\right),\;E_{t}=BERT\;\left(S_{t};\;\theta_{t}^{bert}\right)$

7:  **for** each modality *m*∈{*t*,*a*,*v*} **do**

8:  **if** modality *m* is available then

9:  Feed into Transformer Encoder and infer latent distribution parameters *μ*_*m*_, *σ*_*m*_

10:  Parameterize: $z_{m}=\mu_{m}+\sigma_{m}⊙\epsilon ,\;\epsilon ∼N\left(0,I\right)$

11:  Reconstruct via Transformer Decoder: *h*_*m*_ = *TransformerDecoder*(*z*_*m*_).

12:  **else**

13:  Infer the shared latent variable *z*_*m*_ conditioned on available modalities

14:  Generate the missing modality via conditional decoding: *h*_*m*_ = *TransformerDecoder*(*z*_*m*_, *X*_*avail*_).

15:  **end if**

16:  **end for**

17:  **do** GMFN: *p*_*i*_ = *tanh*(*W*_*i*_*h*_*i*_), *p*_*j*_ = *tanh*(*W*_*j*_*h*_*j*_), *z*_*p*_ = *σ*(*W*_*z*_[*h*_*i*_,*h*_*j*_]), *H*_*ij*_ = *z*_*p*_⊙*p*_*i*_+(1-*z*_*p*_)⊙*p*_*j*_

18:  Aggregate via multi-head attention: *H*_fusion_ = MultiHeadAttention(Concat(*H*_*av*_, *H*_*at*_, *H*_*vt*_)).

19:  Sentiment prediction: $\hat{y}=softmax\;\left(WH+b\right)$

20:  Compute classification loss: $L_{cls}=-\frac{1}{N}\sum_{n=1}^{N}y_{n}\;\log\;{\hat{y}}_{n}$

21:  Compute KL divergence: $L_{KL}=D_{KL}\left(q_{\phi }\left(z|x_{o},\;x_{m}\right)\;\|\;p_{\theta }\left(z|x_{o}\right)\right)$

22:  Compute reconstruction loss: $L_{\text{rec}}=∥x_{m}-{\hat{x}}_{m}{∥}_{2}^{2}$

23:  Joint Optimization Objective Loss: $L=L_{cls}+\lambda_{1}L_{KL}+\lambda_{2}L_{rec}$

24:  Backpropagate L and update parameters

25:  epoch ← epoch – 1

26: **end while**

**Output**: Predicted emotion label $\hat{y}$, probability distribution $\hat{P}$, and the model parameters Θ

Compared with MMIN, GCNet, LNLN, and deterministic Transformer-based reconstruction models, RMM-TCVAE provides a probabilistic reconstruction framework that explicitly models latent uncertainty under missing-modality conditions and combines the reconstructed representations through reliability-aware gated fusion. The effectiveness of RMM-TCVAE can be attributed to the complementary roles of its three core components. First, the TRN captures long-range cross-modal dependencies and contextual relationships, enabling more robust reconstruction of missing-modality representations. Second, the TCVAE module introduces probabilistic latent modeling that explicitly captures uncertainty and preserves semantic consistency across modalities, rather than treating missing-modality recovery as a deterministic mapping. Third, the GMFN dynamically adjusts modality contributions according to feature reliability, allowing the framework to exploit complementary information while reducing the influence of noisy or imperfectly reconstructed features. These mechanisms explain why RMM-TCVAE maintains stable recognition performance, particularly under higher missing-rate settings. The ablation results further confirm that each component contributes substantially to the overall performance gains.

Beyond benchmark evaluations, the proposed framework has practical implications for real-world MER tasks, where complete multimodal observations are often unavailable due to sensor malfunctions, communication interruptions, privacy constraints, environmental noise, or user-related factors. Such incomplete-modality conditions are common in intelligent tutoring systems, affective dialogue agents, telemedicine platforms, social robots, online meeting analysis systems, and human-computer interaction applications. Overall, these findings suggest that probabilistic reconstruction combined with reliability-aware fusion provides a promising direction for robust MER under incomplete-modality conditions.

From a computational perspective, the additional overhead of RMM-TCVAE is mainly associated with the conditional reconstruction and reliability-aware fusion processes. The current runtime and memory analyses indicate that these additional operations remain computationally manageable in the tested GPU-based environment. In the present study, the primary objective is to validate the robustness and computational feasibility of RMM-TCVAE under controlled GPU-based conditions. However, real-time performance on resource-constrained mobile or embedded devices remains to be systematically evaluated. Future work will therefore investigate lightweight reconstruction architectures, model compression, quantization, and other efficient inference strategies to further reduce latency and memory requirements for deployment on mobile and embedded platforms.

### Limitations of the study

Although RMM-TCVAE demonstrates strong performance across multiple benchmark datasets and missing-modality settings, several limitations remain. First, the Transformer Reconstruction Network and conditional variational inference mechanism introduce additional computational costs and memory requirements, which may limit deployment in resource-constrained or real-time applications. Second, the evaluation was conducted on CMU-MOSI, CMU-MOSEI, and IEMOCAP, which provide standardized protocols but may not fully represent the complexity of real-world multimodal environments. Third, the current framework assumes that at least one informative modality is available during inference; when multiple modalities are simultaneously corrupted or contain weak emotional cues, reconstruction quality and recognition performance may decline. Fourth, the present study evaluates missing rates up to 50%, leaving more extreme missing-modality conditions unexplored. Future research should investigate more realistic missing-modality mechanisms, direct reconstruction-quality assessment, and deployment-oriented evaluation under resource-constrained conditions. For more severe missing conditions, including missing rates beyond the current 50% setting, severity-adaptive training could be explored to improve robustness as information loss increases. In addition, when the remaining modalities themselves contain weak or noisy emotional cues, posterior uncertainty estimated by the conditional variational module could be more explicitly incorporated into the fusion process to reduce the influence of low-confidence reconstructed representations.

## Resource availability

### Lead contact

Requests for further information and resources should be directed to and will be fulfilled by the lead contact, Shuai Wang (shuai.wang@xjust.edu.cn).

### Materials availability

This study did not generate new materials.

### Data and code availability

• **Data**: The datasets analyzed in this study are available from their original providers subject to the corresponding access conditions. Access information is listed in the key resources table.
• **Code**: All original code has been deposited via GitHub URL at https://github.com/WANGShuaiShine/RMM-TCVAE and is publicly available as of the date of publication.
• **Additional information**: Additional information required to reanalyze the data is available from the lead contact upon request.

## Acknowledgments

The authors gratefully acknowledge the financial support from the 10.13039/501100003398Shanxi Scholarship Council of China (grant no 2025202), the Fundamental Research Program of Shanxi Province, China (grant no 202403021222304), the Scientific Research and Innovation Team of Data Mining and Industrial Intelligent Application of Yuncheng University (grant no YCXYTD-202402), the Industry-University Research Cooperation Project of Yuncheng University (grant no 202412E101).

## Author contributions

S.W. is responsible for conceptualization, methodology, algorithm implementation, model construction, experiment validation, data collection and processing, formal analysis, software development, visualization, writing, review and editing of the manuscript. J.L. and Z.W. participated in data analysis, result discussion, and manuscript revision. B.L.W. and Q.W. provided resources, project administration, and supervision. All authors have read and approved the final version of the manuscript.

## Declaration of interests

The authors declare no competing interests.

## Declaration of generative AI and AI-assisted technologies in the writing process

During the preparation of this work, the authors used ChatGPT and Grammarly in order to improve language and readability. After using these tools/services, the authors reviewed and edited the content as needed and take full responsibility for the content of the published article.

## STAR★Methods

### Key resources table

**Table:** 

REAGENT or RESOURCE	SOURCE	IDENTIFIER
**Deposited data**		

CMU-MOSI and CMU-MOSEI datasets	CMU-MultiComp-Lab	Available at: https://github.com/CMU-MultiComp-Lab/CMU-MultimodalSDK
IEMOCAP dataset	SAIL Lab, University of Southern California	Available at: https://sail.usc.edu/iemocap/
COVAREP	COVAREP Team	Available at: https://covarep.github.io/covarep
OpenSMILE	audEERING	Available at: https://audeering.github.io/opensmile
FACET	iMotions	Available at: https://imotions.com
Missing Modality Imagination Network (MMIN)	Zhao et al.^13^	Available at: https://github.com/AIM3-RUC/MMIN
IF-MMIN	Zuo et al.^14^	Available at: https://github.com/ZhuoYulang/IF-MMIN
Multimodal Cyclic Translation Network (MCTN)	Pham et al.^15^	Available at: https://github.com/hainow/MCTN
Graph Complete Network (GCNet)	Lian et al.^17^	Available at: https://github.com/zeroQiaoba/GCNet
Language-dominated Noise-resistant Learning Network (LNLN)	Zhang et al.^18^	Available at: https://github.com/Haoyu-ha/LNLN
Noise Imitating-based Adversarial Training (NIAT)	Yuan et al.^19^	Available at: https://github.com/Columbine21/NIAT
Gradient-guided Modality Decoupling (GMD)	Wang et al.^22^	Available at: https://github.com/HaoWang420/Gradientguided-Modality-Decoupling
RMM-TCVAE	This study	Available at: https://github.com/WANGShuaiShine/RMM-TCVAE

**Software and algorithms**		

Python	Python Software Foundation	Version 3.10; https:/www.python.org
PyTorch	PyTorch Team	Version 2.2.0; https:/pytorch.org
Transformers	Hugging Face	Version 4.28.1; https://huggingface.co/docs/transformers
NumPy	NumPy Developers	Version 1.24.3; https://numpy.org
Pandas	Pandas Development Team	Version 2.0.3; https://pandas.pydata.org
Torchvision	PyTorch Team	Version 0.17.2; https://pytorch.org/vision
OpenCV	OpenCV Team	Version 4.9.0.80; https://opencv.org
Matplotlib	Matplotlib Development Team	Version 3.8.4; https://matplotlib.org
PyTorch Grad-CAM	Gildenblat et al.	Version 1.5.2; https://github.com/jacobgil/pytorch-grad-cam
ONNX	ONNX Community	Version 1.16.0; https://onnx.ai
ONNX Runtime GPU	Microsoft	Version 1.17.1; https://onnxruntime.ai
Scikit-learn	Scikit-learn Developers	Version 1.3.0; https://scikit-learn.org
CUDA Toolkit 12.1	NVIDIA	Version 12.1; https://developer.nvidia.com/cuda-toolkit
cuDNN 8.9	NVIDIA	Version 8.9; https://developer.nvidia.com/cudnn

**Other**		

NVIDIA GeForce RTX3090 GPU	NVIDIA	Hardware used for model training and evaluation

### Experimental model and study participant details

#### Datasets

We conducted experiments on the following datasets. Table 8 presents the key statistics and evaluation protocols of the datasets used in this study.

##### CMU-MOSI

CMU-MOSI consists of 2,199 short video segments collected from 93 YouTube movie reviews, each labeled with continuous sentiment intensity scores ranging from −3 (highly negative) to +3 (highly positive). It provides three aligned modalities: transcribed text, visual frames, and acoustic features. To maintain consistency with prior studies, we used the official dataset split.

##### CMU-MOSEI

CMU-MOSEI contains 22,856 annotated utterances from more than 3,225 videos, covering diverse contexts, accents, speaking styles, and recording conditions. The dataset provides aligned text, audio, and visual modalities. Similar to MOSI, each segment is originally annotated with a sentiment intensity score ranging from −3 to +3. MOSEI addresses the limitations of its predecessor MOSI through significantly larger scale, greater speaker diversity, and more balanced sentiment distribution. We followed the standard benchmark split used in prior work to enable fair comparison. For both CMU-MOSI and CMU-MOSEI, a binary classification scheme was applied, where samples with sentiment scores above 0 were treated as positive and those below 0 were treated as negative. Neutral samples (score = 0) are excluded to ensure consistency with standard evaluation protocols.

##### IEMOCAP

IEMOCAP consists of approximately 12 hours of multimodal recordings produced by five groups of trained actors engaged in scripted and improvised dyadic interactions. The dataset provides aligned textual, acoustic, and visual modalities with emotion annotations assigned by multiple human evaluators. The commonly used emotion categories include happiness, anger, sadness, and neutral (4-class), with extended versions incorporating additional labels such as excited and frustrated (6-class), as summarized in Table 8. In this work, experiments were performed under both the 4-class and 6-class settings. Following prior work, we adopted the widely used 5-fold speaker-independent cross-validation (CV) protocol to avoid speaker leakage and ensure fair evaluation. Specifically, in each fold, utterances from one session are used as the test set, while the remaining four sessions are used for training. Final results were obtained by averaging performance across all five folds.

All analyses in this study were conducted using previously released benchmark datasets (CMU-MOSI, CMU-MOSEI, and IEMOCAP); no new participants were recruited and no new human-subject data were collected. Samples were allocated according to the official CMU-MOSI and CMU-MOSEI dataset splits or the five-fold speaker-independent session-based protocol for IEMOCAP, as summarized in Table 8. Participant demographic variables were not used for model training, stratification, or subgroup analysis in the present study. Accordingly, the influence of sex or gender on model performance was not assessed. The original participant recruitment, consent, and institutional oversight procedures are described by the respective dataset providers, and all datasets were used subject to their original access conditions.

#### Implementation details

##### Unimodal feature extraction

For each modality, features were extracted independently from the respective datasets. All modality representations were aligned at the utterance level based on the official dataset annotations to ensure cross-modal consistency. We followed standard feature extraction pipelines widely adopted in prior work for each dataset to ensure fair comparison.

###### Acoustic features

The COVAREP^51^ toolkit was used to extract 74-dimensional frame-level acoustic representations for MOSI and MOSEI, while OpenSMILE^52^ was employed to extract 100-dimensional acoustic features for IEMOCAP using standard configurations widely adopted in prior emotion recognition research.

###### Visual features

Visual representations were obtained using FACET (a commercial facial expression analysis toolkit), resulting in 47-dimensional vectors for MOSI and 35-dimensional vectors for MOSEI. For IEMOCAP, 512-dimensional visual features were extracted from OpenFace 2.0.^53^

###### Textual features

For MOSI and MOSEI, textual features were derived from the pre-trained BERT model.^54^ We used the BERT-base-uncased variant, which comprises 12 Transformer layers with 768-dimensional hidden states and 12 self-attention heads. The final-layer [CLS] embedding was used as the utterance-level representation, producing a 768-dimensional feature vector. For IEMOCAP, 768-dimensional textual representations were obtained using Sentence-BERT,^55^ following prior work to capture sentence-level semantic information in emotion recognition tasks.

##### Experimental setup

To simulate real-world scenarios with incomplete multimodal inputs, we constructed multimodal datasets with artificially introduced missing values. Following the previous works,^17^^,^^18^ missing values were simulated by randomly masking elements within each modality sequence. Specifically, missing tokens in the textual modality were replaced with the placeholder [UNK], while missing segments in the audio and visual modalities are zero-padded. Following Li et al.,^23^ we adopted a fixed missing rate ranging from 0.1 to 0.5 with a step size of 0.1, and evaluated the model under each setting independently. For example, 10% of the information is randomly omitted from each modality within the test set when the missing rate r=0.1. For each modality, the missing rate is predefined and kept consistent across the training, validation, and test sets, in line with prior work.^17^^,^^56^ This setting reflects intra-modality information loss, allowing us to evaluate the robustness of the model under partial corruption conditions. We also evaluated modality-level missing scenarios, where one or more entire modalities are completely unavailable. Together, these settings provide a comprehensive evaluation of the model under generalized incomplete multimodal conditions.

The simulation strategy aims to ensure a controlled and reproducible evaluation of model robustness under incomplete multimodal conditions. Random masking is adopted to approximate the uncertainty and variability of real-world missing patterns, without assuming a fixed distribution. The missing rate is varied from 0.1 to 0.5 to reflect different levels of information loss, from mild to severe. A consistent missing-rate protocol is applied across training, validation, and test sets to ensure fair comparison.

The experimental hyperparameters are summarized in Table 9. During training, we used the AdamW optimizer to update the network parameters. The learning rate for the pre-trained BERT encoder was set to 5×10^-5^, while that for all other modules was 1×10^-4^. The batch size was set to 32 for CMU-MOSI and CMU-MOSEI and 64 for IEMOCAP, and all models were trained for 200 epochs. The loss weights in Equation 19 were set to λ_1_=0.1 and λ_2_=0.01. All experiments were implemented in PyTorch and conducted on an NVIDIA GeForce RTX 3090 GPU with Python 3.10. A dropout rate of 0.1 was applied to all Transformer layers. We applied warm-up scheduling, cosine annealing, and early stopping based on validation performance. To ensure robustness, experiments on MOSI and MOSEI were conducted using three different random seeds, and results were reported as averages. For IEMOCAP, 5-fold speaker-independent cross-validation was adopted. Within each fold, experiments were repeated using three different random seeds, and final results were averaged across folds and seeds.

#### Evaluation metrics

To evaluate the performance of RMM-TCVAE against previous models, we adopt Accuracy (Acc) and Weighted Average F1-score (WAF) as evaluation metrics for a fair comparison. Accuracy is defined as the ratio of correctly predicted samples to the total number of samples:

(Equation 2) $$\begin{array}{c} \text{Acc}=\frac{TP+TN}{TP+TN+FP+FN} \end{array}$$

where *TP*, *TN*, *FP*, and *FN* denote true positives, true negatives, false positives, and false negatives, respectively. The F1-score is defined as the harmonic mean of precision and recall:

(Equation 3) $$\begin{array}{c} F1=\frac{2\cdot \text{Precision}\cdot \text{Recall}}{\text{Precision}+\text{Recall}} \end{array}$$

where

(Equation 4) $$\begin{array}{c} \text{Precision}=\frac{TP}{TP+FP},\text{Recall}=\frac{TP}{TP+FN} \end{array}$$

Given the class imbalance in MOSEI and IEMOCAP,^50^ we additionally report the WAF, defined as:

(Equation 5) $$\begin{array}{c} \text{WAF}=\sum_{c=1}^{C}w_{c}\cdot F1_{c} \end{array}$$

where *C* denotes the number of classes, *F*1_*c*_ is the F1-score of class *c*, and *w*_*c*_ represents the proportion of samples belonging to class *c*:

(Equation 6) $$\begin{array}{c} w_{c}=\frac{N_{c}}{\sum_{i=1}^{C}N_{i}} \end{array}$$

with *N*_*c*_ representing the number of samples in class *c*. This weighted formulation ensures that minority classes retain appropriate influence during evaluation, thereby yielding a more faithful assessment of overall model performance.

### Method details

#### Problem formulation

We aim to design a robust learning framework capable of modeling joint representations under missing-modality conditions for MER. We denote the multimodal input sample as *X*=[*X*_*a*_,*X*_*v*_,*X*_*t*_], where *X*_*a*_, *X*_*v*_, and *X*_*t*_ represent the acoustic, visual, and textual modalities, respectively. To simulate real-world incomplete-modality scenarios, we randomly mask a subset of modalities during the training phase. The masked sample is denoted as $X_{n}^{\prime }$, where *n*∈*a*,*v*,*t* indicates the modality being masked. This strategy enables the model to learn robust cross-modal representations under diverse missing configurations. As illustrated in Figure 8, the dataset includes one full-modality setting and six missing-modality conditions (Conditions 1–6), covering both single-modality absence (e.g., missing visual modality) and multi-modality missing scenarios.

Our goal is to leverage the available multimodal signals to predict the sentiment label of each video segment. For each sample, the low-level feature sequences from textual (*t*), acoustic (*a*), and visual (*v*) modalities may be partially missing due to random masking. These are represented as Equation 7:

(Equation 7) $$\begin{array}{c} U_{t}^{\prime }\in R^{T\times d_{t}},U_{a}^{\prime }\in R^{T\times d_{a}},U_{v}^{\prime }\in R^{T\times d_{v}} \end{array}$$

where *T* denotes the sequence length and *d*_*t*_, *d*_*a*_, *d*_*v*_ denote the feature dimensions of text, audio, and visual modalities, respectively.

During training, the complete modality features $U_{t}\in R^{T\times d_{t}},U_{a}\in R^{T\times d_{a}},U_{v}\in R^{T\times d_{v}}$ along with a modality-missing position indicator matrix are used as inputs. The indicator matrix specifies which modalities are absent and guides the model to learn robust joint representations under incomplete-modality conditions. Finally, the model outputs the predicted sentiment label ${\hat{y}}_{n}$ for each sample.

In this work, we consider a generalized incomplete multimodal learning setting, where input data may be missing in two forms: (1) modality-level missing, where one or more entire modalities are absent; and (2) feature-level missing, where partial information within each modality is corrupted or unavailable. These two types of missingness frequently co-occur in practical applications and pose substantial difficulties for multimodal representation learning. Therefore, our goal is to develop a unified framework capable of handling both types of missing conditions in a robust and consistent manner.

#### Overview of RMM-TCVAE

To address MER under missing-modality conditions, we propose a Transformer-based Conditional Variational Autoencoder framework, termed RMM-TCVAE. As illustrated in Figure 9, the overall architecture consists of five main components: (1) data preprocessing, (2) contextual feature representation, (3) Transformer-based conditional variational reconstruction network, (4) gated multimodal fusion network, and (5) sentiment classifier. For clarity, we describe the framework using the visual modality missing scenario as an example. The overall procedure of the proposed RMM-TCVAE framework is presented in Algorithm 1.

##### Data preprocessing

The raw multimodal inputs are first transformed into modality-specific sequential representations, including textual, acoustic, and visual feature streams. This step ensures standardized inputs for subsequent feature extraction and cross-modal modeling. Each modality is processed into time-aligned feature sequences suitable for temporal representation learning.

##### Contextual feature representation

To capture modality-specific contextual information, we employ dedicated encoders for each modality. The textual modality is encoded using a pre-trained Bidirectional Encoder Representations from Transformers (BERT)^54^ model, and the sentence-level contextual embedding is denoted as *E*_*t*_*.* Regarding acoustic and visual input, we adopt single-layer Long Short-Term Memory networks (sLSTM) to learn deep temporal dependencies, producing representations *E*_*a*_ and *E*_*v*_, respectively. These contextual embeddings serve as high-level modality-specific representations for subsequent generative modeling.

##### Transformer-based conditional variational reconstruction network

The encoded modality features are then fed into a Transformer-based CVAE (TCVAE). This module comprises a Transformer encoder, a latent variable inference layer, and a Transformer decoder. The Transformer encoder captures cross-modal contextual dependencies, while the latent space models the shared probabilistic structure among modalities.

Under missing-modality conditions (e.g., missing visual modality in Figure 9), the model performs conditional latent inference by leveraging the available modalities as guidance signals. Specifically, the latent variable is inferred from observed modalities, and the decoder reconstructs the missing modalities representation accordingly. By conditioning the generative process on existing modality features, the framework reduces semantic ambiguity and improves reconstruction fidelity in incomplete-modality scenarios.

##### Gated multimodal fusion network

Prior to final prediction, a gated attention fusion mechanism is introduced to dynamically integrate complementary information across modalities. Unlike static fusion strategies, the gated module adaptively reweights modality contributions based on their reliability and informativeness, thereby mitigating feature degradation caused by modality absence. This reliability-aware fusion enhances robustness and stabilizes representation learning under diverse missing configurations.

##### Sentiment classification

The reconstructed modality representations are combined with the originally available modality features to form a unified latent representation. This joint embedding is then passed through a multi-layer perceptron (MLP), followed by a Softmax layer to predict sentiment categories. The end-to-end training objective optimizes both generative reconstruction and discriminative classification, enabling accurate MER across varying missing-modality conditions.

The design of RMM-TCVAE is theoretically motivated by three key requirements of missing-modality MER: cross-modal dependency modeling, uncertainty-aware reconstruction, and reliability-adaptive fusion. First, because emotional cues are jointly expressed through textual, acoustic, and visual signals, missing modality representations should be inferred from both intra-modal context and inter-modal dependencies. The TRN is therefore adopted to capture long-range contextual relations and cross-modal interactions through self-attention. Second, missing or corrupted modalities are inherently uncertain, and a deterministic reconstruction strategy may produce overconfident or semantically inconsistent representations. To address this issue, the TCVAE formulates reconstruction as probabilistic latent inference, allowing the model to represent the distributional uncertainty of missing modality features. Third, observed and reconstructed modalities should not contribute equally to the final prediction because their reliability may differ under incomplete conditions. The GMFN module is therefore introduced to dynamically estimate modality-specific contribution weights and suppress unreliable reconstructed information. Together, these theoretical considerations justify the major architectural components of RMM-TCVAE.

#### Data preprocessing

As shown in Figure 9 (Step ①), raw utterances are first converted into modality-specific numerical sequential representations to enable structured multimodal processing. For each modality, features are extracted either through conventional signal processing techniques or via neural network-based encoders to obtain discriminative deep representations. Comprehensive details regarding the single-modality feature extraction procedures are presented in subsection Unimodal Feature Extraction. After extracting low-level modality features in the preprocessing stage, contextual encoders are applied to obtain higher-level semantic representations.

#### Contextual feature representation

To enable the reconstruction network to obtain sufficiently informative modality-specific representations, we employ dedicated deep learning encoders to transform the extracted features into contextualized embeddings. Specifically, different encoding architectures are adopted for different modalities to capture their intrinsic characteristics and temporal dependencies.

For the textual modality, a pre-trained BERT model is utilized to encode contextual semantic information. For the acoustic and visual modalities, single-layer unidirectional Long Short-Term Memory networks (sLSTM) are adopted to model temporal dynamics and extract deep sequential representations.

As illustrated in Figure 9 (Step ②), the three modalities are processed through their respective encoders to obtain contextual feature representations, which can be formulated as:

(Equation 8) $$\begin{array}{l} E_{m}=sLSTM\left(S_{m};{\theta_{m}}^{lstm}\right)\\ E_{t}=BERT\left(S_{t};{\theta_{t}}^{bert}\right) \end{array}$$

where *m*∈*a*,*v* denotes the acoustic and visual modalities, and *E* represents the encoded feature representation of the three modalities after contextual encoding. These contextual embeddings serve as modality-specific high-level representations for subsequent conditional variational modeling and cross-modal interaction learning.

#### Transformer-based conditional variational reconstruction network (TRN)

We design a Transformer-based Conditional Variational Reconstruction Network (TRN) within the proposed RMM-TCVAE framework. As shown in Figure 9 (Step ③), the reconstruction module consists of two Transformer Conditional Variational Autoencoders (TCVAEs): one TCVAE models the available modalities to learn modality-specific latent representations, while the other TCVAE reconstructs the missing modality conditioned on the learned latent structure and the available modality representations. Each TCVAE contains three key components: a Transformer encoder, a latent space estimation network, and a Transformer decoder.

The proposed framework is modality-agnostic and can flexibly handle arbitrary combinations of available and missing modalities. The visual-missing scenario is used here as an illustrative example. First, the available-modality TCVAE is trained using the textual and acoustic modalities. The available-modality features are fed into the Transformer encoder to infer the parameters of the corresponding modality-specific latent distribution, from which latent variables are subsequently sampled. The sampled latent variable is then passed to the decoder for reconstruction. The parameters of the variational autoencoder are optimized by minimizing the KL divergence loss $L_{\text{KL}}$ and the reconstruction loss $L_{\text{rec}}$, ensuring that the modality-specific latent space accurately captures the intrinsic characteristics of the observed modalities. Subsequently, the reconstruction TCVAE for the visual modality is trained to recover the missing modalities. The incomplete modality input is fed into the reconstruction TCVAE encoder to estimate the parameters of a unified shared latent distribution, from which latent samples are obtained. Meanwhile, the encoded representations from the textual and acoustic TCVAEs are used as conditional inputs to generate conditional representations. These conditional representations are combined with the sampled latent variable and passed into the decoder to reconstruct the missing modalities. The reconstruction TCVAE is optimized by jointly minimizing $L_{\text{KL}}$ and $L_{\text{rec}}$, thereby ensuring the accuracy and semantic consistency of the reconstructed modality representations.

##### Transformer encoder

The Transformer encoder aims to capture contextual dependencies across heterogeneous modalities. Although structurally similar to the standard Transformer architecture, additional modality-type embeddings and positional embeddings are incorporated to distinguish modality characteristics. The input representations corresponding to text and audio modalities are given in Equation 9:

(Equation 9) $$\begin{array}{c} P=\left[x_{t};x_{a}\right]⊕\left[M_{t};M_{a}\right]⊕\left[P_{t};P_{a}\right] \end{array}$$

where *x*_*t*_ and *x*_*a*_ denote the encoded textual and acoustic feature representations input to the Transformer encoder, respectively; *M*_*t*_ and *M*_*a*_ denote the modality-type embeddings used to distinguish different modalities; *P*_*t*_ and *P*_*a*_ denote the corresponding positional embeddings; ⊕ denotes element-wise addition; and [ ; ] denotes concatenation.

The contextualized output of the Transformer encoder is expressed as:

(Equation 10) $$\begin{array}{c} P^{\prime }=TransformerEncoder\left(P\right) \end{array}$$

After *N* stacked Transformer layers, the final encoder output is fed into the latent space estimation network to infer the parameters of the latent distribution.

##### Latent space estimation network

We construct a TCVAE to model the latent distribution of multimodal data and generate missing modality representations through an encoder–decoder architecture. Given the observed modalities *x*_*o*_ and the missing modality *x*_*m*_, the VAE aims to maximize the conditional marginal likelihood *p_θ_* (*x*_*m*_|*x*_*o*_). In practical applications, since the latent variable *z* typically resides in a high-dimensional space, directly computing the integral of the conditional marginal likelihood *p*_*θ*_ (*x*_*m*_|*x*_*o*_) is computationally intractable. Therefore, we introduce a variational posterior distribution *q*_*ϕ*_(*z*|*x*_*o*_,*x*_*m*_) to approximate the intractable posterior distribution and optimize the model through variational inference. By employing gradient-based optimization to maximize the Evidence Lower Bound (ELBO), we obtain the optimal conditional variational approximation *q*_*ϕ*_(*z*|*x*_*o*_,*x*_*m*_).

(Equation 11) $$\begin{array}{c} \log\;p_{\theta }\left(x_{m}|x_{o}\right)\geq E_{q_{\phi }\left(z|x_{o},x_{m}\right)}\left[{\log\;p}_{\theta }\left(x_{m}|z,x_{o}\right)\right]-D_{KL}\left(q_{\phi }\left(z|x_{o},x_{m}\right)\;\|\;p_{\theta }\left(z|x_{o}\right)\right) \end{array}$$

where *x*_*o*_ denotes the observed modalities and *x*_*m*_ denotes the missing modality to be reconstructed. *q*_*ϕ*_ (*z*|*x*_*o*_,*x*_*m*_) represents the conditional approximate posterior distribution, *p*_*θ*_(*z*|*x*_*o*_) denotes the conditional prior distribution, *p*_*θ*_ (*x*_*m*_|*z*,*x*_*o*_) corresponds to the conditional likelihood used for modality reconstruction, and *D*_*KL*_ measures the divergence between the conditional approximate posterior *q*_*ϕ*_ (*z*|*x*_*o*_,*x*_*m*_) and the conditional prior *p*_*θ*_ (*z*|*x*_*o*_). The first term maximizes the reconstruction quality of the missing modality, while the KL-divergence term regularizes the latent distribution to remain close to the conditional prior, thereby improving generalization and robustness under missing-modality conditions.

It is important to note that the latent variable ***z*** is shared across all modalities rather than being modality-specific. This shared latent space is designed to capture the joint emotional semantics that are common to text, audio, and visual signals. Under missing-modality conditions, the approximate posterior *q*_*ϕ*_ (*z*|*x*_*o*_) is conditioned only on the available modalities, yet the latent space itself remains the same across different missing patterns. This design ensures that the reconstructed missing modalities are consistent with the same underlying emotional state, avoiding the fragmentation that would arise from modality-specific latent variables. The conditional generation process *p* (*x*_*m*_∣*z*, *x*_*o*_) then uses this shared latent representation to produce semantically coherent reconstructions.

The fused multimodal representation produced by the Transformer encoder is fed into the TCVAE to estimate the parameters of the conditional latent distribution, effectively transforming features from the original feature space into a probabilistic latent space. Latent variables are then sampled from the learned conditional latent distribution using the reparameterization trick. To enable end-to-end training via gradient descent, the reparameterization trick is applied to generate samples according to the latent distribution. The resulting samples are used as latent representations of the input features.

At the final stage, the sampled latent variable *z* is input into a Transformer-based decoder to perform reconstruction. While structurally analogous to the encoder, the decoder is tailored for generative reconstruction. The training objective jointly minimizes the reconstruction error between inputs and outputs, as well as the KL divergence that enforces alignment between the conditional approximate posterior and the conditional prior. This joint objective enables end-to-end optimization of the entire framework.

##### Transformer decoder

The Transformer decoder generates missing modalities using latent representation inferred from the observed modalities. The input to the decoder consists of the latent variable *z* and the feature representations of the observed modalities augmented with positional information.

Although the decoder shares a similar architectural backbone with the Transformer encoder, it is specifically adapted for generative modeling. The decoder is composed of multiple self-attention layers and feed-forward networks, enabling it to model salient dependencies and progressively refine the generated features.

The input representations pass through stacked decoder layers to gradually reconstruct the missing modalities. At the *i*^th^ layer, the decoder output can be expressed as:

(Equation 12) $$\begin{array}{c} h_{i}=\text{TransformerDecoder}\left(h_{i-1}\right) \end{array}$$

where *h*_*i*_ denotes the output feature of the *i*^*th*^ decoder layer, and *h*_*i*-1_ represents the output from the previous layer.

The decoder outputs reconstructed representations for the missing modalities. To enhance generation quality, a reconstruction loss $L_{\text{rec}}$ is introduced to measure the discrepancy between the generated modality features and the ground-truth modality features. By minimizing this reconstruction loss, the decoder is optimized to produce accurate and semantically consistent representations of the missing modalities.

The motivation behind TRN is that multimodal emotion expressions often exhibit strong semantic correlations across modalities. Even when one modality is missing, the remaining modalities still contain complementary emotional information. By leveraging the self-attention mechanism, Transformer encoders can effectively model long-range cross-modal dependencies and facilitate reconstruction of missing modality representations.

#### Gated multimodal fusion network (GMFN)

Under the supervision of reconstructed modality representations and available modality features, we introduce a Gated Multimodal Fusion Network (GMFN) to adaptively integrate multimodal information. As illustrated in Figure 10, the proposed fusion mechanism learns modality-specific gating weights in a robust manner, enabling dynamic adjustment of modality contributions under missing-modality conditions. Specifically, we first obtain modality representations from the Transformer decoder, denoted as *h*_*m*_, where *m*∈{*a*,*v*,*t*} corresponds to acoustic, visual, and textual modalities, respectively. For each pair of modalities (e.g., *h*_*i*_ and *h*_*j*_), a gated interaction mechanism is applied to compute their fused representation *H*_*ij*_, where *ij*∈{*av*,*at*,*vt*}. The gated fusion process can be formulated as follows:

(Equation 13) $$\begin{array}{l} p_{i}=\tanh\left(W_{i}h_{i}\right)\\ p_{j}=\tanh\left(W_{j}h_{j}\right)\\ z_{p}=\sigma \left(W_{z}\left[h_{i};h_{j}\right]\right)\\ H_{ij}=z_{p}⊙p_{i}+\left(1-z_{p}\right)⊙p_{j} \end{array}$$

where *W*_*i*_, *W*_*j*_, and *W*_*z*_ are trainable weight matrices, with *W*_*z*_ specifically designed to model the relative contribution of each modality within a modality pair. Here, the function σ(·) refers to the sigmoid activation, and ⊙ denotes element-wise product.

The gating vector *z*_*p*_ adaptively controls the contribution of each modality within each pair, guiding the model to assign higher weights to more informative or reliable modalities. After computing pairwise gated representations *H*_*av*_, *H*_*at*_, *H*_*vt*_, they are concatenated and further integrated via the multi-head attention mechanism to obtain the final fused representation:

(Equation 14) $$\begin{array}{c} H_{\text{fusion}}=\text{MultiHeadAttention}\left(C\text{oncat}\left(H_{av},H_{at},H_{vt}\right)\right) \end{array}$$

This hierarchical gated fusion strategy effectively mitigates feature degradation caused by missing modalities and enhances the robustness of multimodal representation learning in MER.

Observed and reconstructed modalities may contribute differently to emotion recognition due to varying information quality and reconstruction uncertainty. Therefore, assigning identical importance to all modalities may amplify noisy representations. The proposed gating mechanism dynamically adjusts modality contributions according to their informativeness, resulting in more reliable multimodal fusion.

#### Sentiment classification

A multimodal Transformer is employed to further integrate the fused multimodal representations. The resulting representation is then processed by a Multi-Layer Perceptron (MLP), followed by a Softmax layer to predict sentiment labels for MER. The prediction function can be expressed as:

(Equation 15) $$\begin{array}{c} \hat{y}=softmax\left(WH+b\right) \end{array}$$

where $\hat{y}$ denotes the predicted probability distribution over sentiment categories, $H\in R^{d}$ represents the fused multimodal feature representation, and *W* and *b* are trainable parameters. The entire framework is optimized in an end-to-end manner to jointly learn feature representations and perform emotion classification under missing-modality conditions.

#### Joint optimization objective loss function

To effectively utilize both complete and incomplete modality data, we design three loss components: the classification loss $L_{\text{cls}}$, the KL divergence loss $L_{\text{KL}}$, and the reconstruction loss $L_{\text{rec}}$. These components are integrated into a unified objective function for end-to-end optimization, as illustrated in Figure 9. The classification loss $L_{\text{cls}}$ is formulated using the cross-entropy criterion:

(Equation 16) $$\begin{array}{c} L_{cls}=-\frac{1}{N}\sum_{n=1}^{N}y_{n}\;\log\;{\hat{y}}_{n} \end{array}$$

where *N* denotes the total number of training instances, *y*_*n*_ represents the ground-truth label of the *n*^th^ sample, and ${\hat{y}}_{n}$ represents the predicted probability.

The KL divergence loss follows the variational formulation:

(Equation 17) $$\begin{array}{c} L_{KL}=D_{KL}\left(q_{\phi }\left(z|x_{o},x_{m}\right)\;\|\;p_{\theta }\left(z|x_{o}\right)\right) \end{array}$$

The reconstruction loss $L_{\text{rec}}$ quantifies the discrepancy between the reconstructed modality representation and its corresponding original feature:

(Equation 18) $$\begin{array}{c} L_{\text{rec}}=∥x_{m}-{\hat{x}}_{m}{∥}_{2}^{2} \end{array}$$

where *m*∈{*a*,*v*,*t*} indicates the modality index, and ${\hat{x}}_{m}$ represents the reconstructed feature generated by the Transformer decoder within the VAE framework.

The proposed training objective can be derived from the ELBO for conditional generative modeling under missing modalities. Let *x*_*o*_ and *x*_*m*_ denote the observed and missing modalities, respectively. Maximizing the conditional log-likelihood *log*⁡*p*(*x*_*m*_∣*x*_*o*_) leads to the ELBO: $E_{q\left(z|x_{o}\right)}\left[\log p\left(x_{m}|z,x_{o}\right)\right]-D_{KL}\left(q\left(z|x_{o}\right)∥p\left(z|x_{o}\right)\right)$. The first term corresponds to the reconstruction loss $L_{\text{rec}}$, and the second to $L_{\text{KL}}$. The classification loss $L_{\text{cls}}$ is added to further enhance discriminative power, resulting in the full objective of Equation 19. The overall objective function is defined as:

(Equation 19) $$\begin{array}{c} L=L_{cls}+\lambda_{1}L_{KL}+\lambda_{2}L_{rec} \end{array}$$

where λ_1_ and λ_2_ are hyperparameters controlling the relative importance of the KL divergence and reconstruction losses, respectively.

By jointly optimizing these three loss components, the proposed RMM-TCVAE framework simultaneously enforces discriminative learning for MER, probabilistic regularization of the latent space, and accurate reconstruction of missing modalities.

#### Baseline models

To rigorously evaluate the proposed RMM-TCVAE framework, we benchmarked it against multiple state-of-the-art methods for MER in the presence of missing modalities, spanning translation-based modeling, imagination-driven reconstruction, graph-structured completion, and noise-robust representation learning paradigms. Specifically, MCTN leverages cyclic cross-modal translation to learn shared representations and serves as a foundational cross-modal alignment baseline, although it was originally designed for complete-modality settings. Reconstruction-based approaches, including MMIN and its invariant-feature extension IF-MMIN, attempt to recover missing modalities through latent imagination mechanisms under predefined missing-modality combinations, yet they do not explicitly address rate-based stochastic missing scenarios. To model structured inter-modal dependencies, GCNet employs graph completion techniques and supports flexible missing-rate configurations, while GMD introduces gradient-guided modality decoupling to enhance robustness under incomplete inputs. In addition, noise-aware learning strategies are represented by NIAT, which integrates noise-intimating adversarial training to mitigate degradation under random and structured missing patterns, and LNLN, which proposes a language-dominated noise-resistant framework capable of handling modality corruption across a wide range of missing rates.

Since previously reported results are often obtained under different experimental configurations, including inconsistent missing-rate settings or dataset splits, direct comparison is not always feasible. Therefore, we re-implemented all baseline models using publicly available source code and evaluated them under a unified training protocol, including identical missing-rate configurations, optimization settings, and metrics.

### Quantification and statistical analysis

Model development, training, and evaluation were conducted using Python 3.10 and PyTorch 2.2.0 on a workstation equipped with an NVIDIA GeForce RTX 3090 GPU. Computations were accelerated with CUDA 12.1 and cuDNN 8.9. Model performance was quantified using Accuracy (Acc) and Weighted Average F1-score (WAF), as defined in Equations 2, 3, 4, 5, and 6. For CMU-MOSI and CMU-MOSEI, the official dataset splits were used, with n representing the number of utterance-level samples in the test set (*n* = 686 for CMU-MOSI and *n* = 4,659 for CMU-MOSEI). For IEMOCAP, five-fold speaker-independent cross-validation was adopted. The total evaluated utterances were 5,531 for the four-class setting and 7,433 for the six-class setting. Experiments on CMU-MOSI and CMU-MOSEI were repeated using three random seeds, and the reported values represent the average performance across repeated runs. For IEMOCAP, each fold was repeated using three random seeds, and final results were averaged across folds and seeds. No inferential statistical tests were conducted because the study focuses on benchmark performance comparison rather than hypothesis testing. Therefore, no *p* values or statistical significance markers are reported. Runtime and memory consumption were measured on the CMU-MOSEI dataset using the same batch size of 32 as reported in Table 9. Training time denotes the average wall-clock time per training epoch, and inference time denotes the average runtime required to process one test sample. Visual materials, including the graphical abstract and experimental figures, were produced with Python-based visualization tools.
